# Interconversion of Functional Motions between Mesophilic and Thermophilic Adenylate Kinases

**DOI:** 10.1371/journal.pcbi.1002103

**Published:** 2011-07-14

**Authors:** Michael D. Daily, George N. Phillips, Qiang Cui

**Affiliations:** 1 Department of Chemistry, University of Wisconsin – Madison, Madison, Wisconsin, United States of America; 2 Computation and Informatics in Biology and Medicine Training Program, University of Wisconsin – Madison, Madison, Wisconsin, United States of America; 3 Departments of Biochemistry and Computer Sciences, University of Wisconsin – Madison, Madison, Wisconsin, United States of America; 4 Theoretical Chemical Institute, University of Wisconsin – Madison, Madison, Wisconsin, United States of America; National Cancer Institute, United States of America and Tel Aviv University, Israel

## Abstract

Dynamic properties are functionally important in many proteins, including the enzyme adenylate kinase (AK), for which the open/closed transition limits the rate of catalytic turnover. Here, we compare our previously published coarse-grained (double-well Gō) simulation of mesophilic AK from *E. coli* (AKmeso) to simulations of thermophilic AK from *Aquifex aeolicus* (AKthermo). In AKthermo, as with AKmeso, the LID domain prefers to close before the NMP domain in the presence of ligand, but LID rigid-body flexibility in the open (O) ensemble decreases significantly. Backbone foldedness in O and/or transition state (TS) ensembles increases significantly relative to AKmeso in some interdomain backbone hinges and within LID. In contact space, the TS of AKthermo has fewer contacts at the CORE-LID interface but a stronger contact network surrounding the CORE-NMP interface than the TS of AKmeso. A “heated” simulation of AKthermo at 375K slightly increases LID rigid-body flexibility in accordance with the “corresponding states” hypothesis. Furthermore, while computational mutation of 7 prolines in AKthermo to their AKmeso counterparts produces similar small perturbations, mutation of these sites, especially positions 8 and 155, to glycine is required to achieve LID rigid-body flexibility and hinge flexibilities comparable to AKmeso. Mutating the 7 sites to proline in AKmeso reduces some hinges' flexibilities, especially hinge 2, but does not reduce LID rigid-body flexibility, suggesting that these two types of motion are decoupled in AKmeso. In conclusion, our results suggest that hinge flexibility and global functional motions alike are correlated with but not exclusively determined by the hinge residues. This mutational framework can inform the rational design of functionally important flexibility and allostery in other proteins toward engineering novel biochemical pathways.

## Author Summary

Dynamic properties are functionally important in many proteins, including the enzyme adenylate kinase (AK), which undergoes chemically rate-limiting domain motions coupled to substrate binding. Since mesophiles and thermophiles often differ in functionally important motions, we compare coarse-grained simulations of AKmeso and AKthermo as well as several proline and glycine mutational variants designed to interconvert the dynamics. As might be expected, both domain motions and local unfolding motions are reduced in AKthermo relative to AKmeso. In AKthermo, both of these types of motions can be partially shifted toward more flexible AKmeso by heating or by mutating hinge prolines. However, only mutation to highly flexible glycine produces motions like those of AKmeso. Thus, the rate-limiting global transition likely depends on a combination of hinge flexibility and stability within the LID and NMP domains. Finally, this mutagenic framework can inform the rational design of flexibility and allostery in other proteins toward engineering novel biological control systems.

## Introduction

Many recent works have provided evidence that not just the average structure, but also motions or “dynamics” around this structure, are important to protein functions including catalytic rate control [1]-[4], macromolecular recognition [5], and/or allosteric regulation [6]–[9]. For therapeutic and engineering applications, it is important to understand physical allosteric mechanisms in specific proteins [10]. Recent studies have been building evidence to support the hypothesis that evolution has selected well-defined motions in allosteric proteins. For example, motions in apo-proteins tend to parallel closure pathways associated with ligand binding [11]–[13]. In addition, dynamics at different timescales appear to be intimately linked [14]. Detailed experimental and computational characterizations often reveal surprising results. For example, transition path sampling and free energy calculations for CheY have revealed a mechanism intermediate between the conformational selection and induced fit extremes [15], [16]. Furthermore, in enzymes with lid-gated active sites, the closure transition is likely to follow a different pathway in the absence vs. the presence of ligand [17]–[19].

Allosteric-type proteins with mesophilic and thermophilic homologues provide useful model systems for investigating the sequence and structural basis of functional motions and for redesigning allostery. According to the “corresponding states” hypothesis [20], evolution maintains a similar functionally competent conformational ensemble at each organism's optimal temperature, and some of the sequence differences between homologues relate to the control of functional motions. Homologous mesophile-thermophile pairs for IPMDH [21] and GAPDH [22] have similar flexibilities at functionally important regions at optimal temperatures separated by 20–40°C. In alcohol dehydrogenase, only a few functionally important regions maintain corresponding states [23], and in other proteins, the thermophile as a whole is *more* flexible than the mesophile [24], [25]. In adenylate kinase, the large CORE domain determines the global stability difference between a mesophile and a thermophile, while the smaller LID and NMP domains are responsible for differencmes in catalytically important dynamics [26].

In this work, we compare conformational transition simulations of homologous mesophilic and thermophilic adenylate kinases and several mutational variants. Crystal structures of apo and bound adenylate kinase (AK) show that upon substrate binding, two small domains (LID and NMP) close over the larger CORE domain; that is, the two predominant states are open (O) and closed (C) [27]. AK recycles AMP to ADP by phosphorylating it with ATP. The substrates ATP and AMP bind at the CORE-LID and CORE-NMP interfaces, respectively. NMR experiments have shown that in thermophilic adenylate kinase from *Aquifex aeolicus* (AKthermo), the six-times-slower opening rate relative to *E. coli* AK (AKmeso) limits the catalytic turnover rate at room temperature [3]. This conformational gating of a chemical reaction is analogous to allostery, though it is important to note that this gating is not equivalent to catalysis of the chemical step by the conformational process [28]. Swapping the entire LID and NMP domain sequences, not just the CORE-LID hinges, between homologous *Bacillus* mesophilic and thermophilic AKs interconverts the catalytic properties (and thus presumably the O/C rate) [26]. Experiments show that the LID and NMP domains and their hinges with CORE are both more stable [29], less functionally susceptible to fragmentation of the primary structure [30], and less flexible at the ps/ns timescale [14] in AKthermo than in AKmeso.

Short (10-ns) atomistic molecular dynamics (MD) simulations of an AKthermo computational mutant suggest that some of the hinge prolines unique to AKthermo limit functionally important ps/ns dynamics at room temperature, and that heating to 350K reduces the order parameters toward those observed for more flexible AKmeso [14]. Our previous double-well coarse-grained simulation of AKmeso [18], which could assess local unfolding and µs-ms conformational transition dynamics that are beyond the reach of standard atomistic simulations, identified several loops likely to be important to the opening (C to O) and/or closing (O to C) transitions. These loops do not exhibit cracking [18], that is, a greater degree of small local unfolding (∼30°) in the transition state than in the open state, as previously hypothesized [31].

In this work, we simulate double-well coarse-grained models of AKthermo and several variants of AKmeso and AKthermo and compare to our AKmeso results according to our previous analytical framework [18]. In this framework, the closing and opening rates are influenced by the relative entropic and enthalpic characteristics of O, C, and transition state (TS) simulation sub-ensembles. Entropy and enthalpy correspond to backbone unfolding and contact formation, respectively, in the current simulation context, and approximately in the physical context. Enthalpic and entropic changes among states have been increasingly recognized as important for classifying allosteric proteins and understanding their mechanisms [32]. In addition, a Gō-like model that permits local unfolding predicts a faster O/C conformational transition rate for AK than does a model with harmonic contact potentials [33] similar to the harmonic representation of residue contacts in the elastic network model [34].

To approximately assess the correspondence of ensembles [20] between AKmeso and AKthermo at their optimal temperatures, we simulate AKthermo at a higher temperature of 375K, where it has similar stability as AKmeso does in simulations at 300K. To assess the importance of hinge flexibility to the global transition, we simulate several mutants of AKthermo at 300K with varying hinge flexibility. These mutants will address seven proline sites, including the four hinge prolines mutated by Henzler-Wildman et al. [14]. AKthermo-7P will mutate these sites to the presumably more flexible corresponding residues from AKmeso, and AKthermo+7G will mutate these sites to glycines, which will theoretically maximize hinge flexibility. AKmeso+7P will mutate the 7 sites in AKmeso to presumably less flexible proline. We also simulate individual mutants as necessary to isolate the origins of differential behaviors of the 7-site mutants relative to wild type.

For conformational transition simulations, double-well coarse-grained (CG) models provide a useful first approximation. While they lack the rigor of atomistic simulations [35], [36], CG approaches can simulate large-scale conformational transitions without constraints so that the trajectory can be meaningfully projected onto any arbitrary reaction coordinate representing a particular motion in Cartesian (rigid-body), backbone dihedral, or contact space. We employ a macroscopic double-well Gō model from Best et al. [37], which mixes single-well potentials based on the native contacts of the O and C crystal structures. According to one interpretation, coarse-grained models such as the double-well Gō model successfully capture gross features of large-scale structural transitions in proteins because structural topology determines the global properties of biomolecules [38]–[40].

We first describe the key sequence and structural differences between AKmeso and AKthermo. Several key analyses will illuminate functionally important differences among AKmeso and AKthermo simulations and their variants. Changes in global motions are assessed by the distribution of states in the two-dimensional energy landscape along CORE-NMP and CORE-LID rigid-body reaction coordinates. Changes in conformational entropy are revealed by comparing residue folding probabilities in regions that make important dynamic contributions in AKmeso [18], including several loops and the flexible LID domain. Approximate enthalpic effects of perturbations are assessed by comparing contact probabilities in O, TS, and C ensembles between two simulations.

## Results

### Setup

#### Key sequence and structural differences between AKmeso and AKthermo

**Figure 1:**
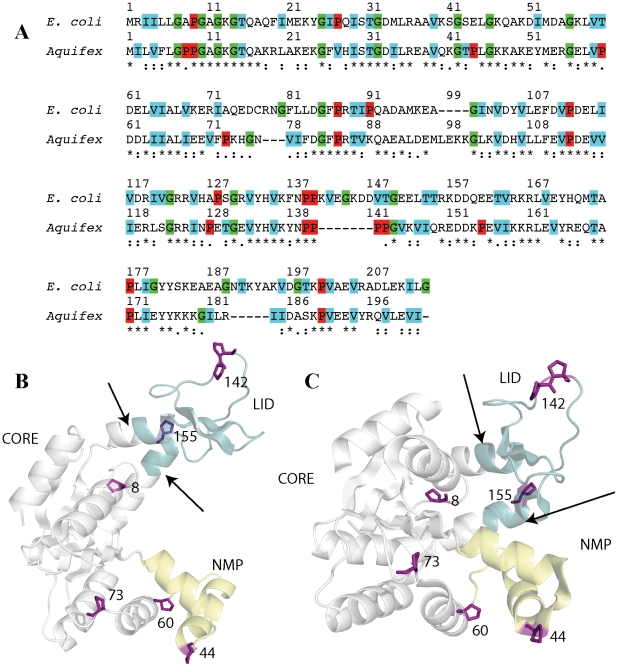
Sequence and structural alignments of AKmeso and AKthermo. A: Sequence alignment between AKmeso and AKthermo performed by clustalw [41]. Red indicates proline, cyan indicates β-branched amino acids (T, I, V), and green indicates glycines. (B) and (C) indicate the positions of the seven hinge prolines (position 143 is omitted for clarity) on the AKmeso open and closed structures (2RH5 [42] and 2RGX [42], respectively).

Figure 1A shows the sequence alignment of AKmeso and AKthermo, which are 47% identical, based on clustalw [41]. One loop (residues 97–100) is present only in AKthermo, and three sequences (residues 141–147 within the LID and loops 78–80 and 190–194) are unique to AKmeso. In addition, as noted in Henzler-Wildman et al. [14], AKthermo has 7 unique prolines while AKmeso has only 2, not counting AKmeso-P128 and AKthermo-P128, which are only one aligned position apart. Given the 47% sequence identity, not all of the 7 unique prolines may be important for modulating functional motions. Of the AKthermo-unique prolines, four (P44, P60, P73, and P155) occur at hinges between secondary structure elements and/or domains, P8 is near an ATP-binding loop and spatially near the CORE-LID connector helices, and P142–143 in AKthermo extends a P_2_ sequence in AKmeso to a P_4_ sequence. By contrast to proline, almost all glycines, which are expected to increase local flexibility, are conserved between AKmeso and AKthermo. The structural positions of these hinges in AKthermo and AKmeso are indicated in Figure 1, panels B and C.

Figure S1, panels A and B superimpose AKmeso (blue) and AKthermo (orange) structures in the open and closed states, respectively, based on equivalent residues in the CORE. As noted by Henzler-Wildman et al. [42], the open states are very similar structurally, with LID being slightly more open in AKmeso. In addition, we note that LID and NMP center of mass positions relative to the CORE vary by a few Ångstroms among O crystal structures of homologous AKs [43]. By contrast, the closed structures show some possibly important differences. In AKthermo, the CORE is unsurprisingly more compact than in AKmeso, but NMP and especially LID are *less* fully closed upon the CORE than in AKmeso. This suggests that the sequence differences between AKmeso and AKthermo may impact domain-scale structure and/or fluctuations in the closed state as well as local flexibility.

#### Parameterization of the AKthermo model

The AKthermo Gō model uses a similar setup as our previous AKmeso model [18]; we describe key differences briefly here and in more detail in the methods. For example, to simulate the stabilization of the closed state by ligand binding, we add selected ligand-mediated interactions to the AKthermo C potential. Table S1 gives the exponential averaging parameters [37] for calibrating the relative stability the of O and C (***ε***_O_) and the transition rate (*β*_mix_) for each simulation. We also use the same absolute contact energy scale as in the AKmeso model so that equivalent pairs of interacting residues have identical energies in both systems. For the simulation to test the corresponding states hypothesis [20] in AKthermo, we choose a simulation temperature of 375K to approximately match the stability of AKmeso at 300K. We calibrate the relative population of C to O to about 4 for all AKmeso and AKthermo simulations based on the estimated experimental *K*_eq_ for AKmeso [3] to acquire good statistics on both O and C ensembles and because simulation results are relatively insensitive to the calibrated *K*_eq_. Furthermore, to identify kinetic properties from a given simulation, we partition the trajectory structures into O, C, and TS ensembles as before [18] based on the Hummer criterion [44], [45]. By this criterion, a TS ensemble, which also marks the boundary between O and C ensembles, should lie in a region of the global contact-based reaction coordinate(s) space that is optimally selective for “reactive” configurations that lie along paths that cross between O and C wells without re-crossing.

#### Mutant initial conformation preparation

To limit the creation of artifacts by the preparation of the mutants' initial conformations, we assess two different minimization protocols by comparing Gō simulation results between wild-type simulations based on the contacts of the crystal structures vs. the contacts of the minimized crystal structures. As the methods detail, we use position-restrained minimization, in which all residues but the mutation sites and closely contacting residues are restrained, because this protocol perturbs the results of the wild-type crystal structure-based simulations much less than unrestrained minimization. In addition, for consistency of simulation comparisons, we use the Gō simulations based on minimized wild type AKmeso and AKthermo, rather than those based on the crystal structures, as the reference data.

### Cartesian space; LID and NMP rigid-body motions

For each simulation,Table S2 shows the average distance of the O and C wells from the O and C crystal structures, respectively, in C_α_ root mean square distance (*rms*) and *Q* space. The rmsd values (typically 1.5–2.5 Å) are reasonable deviations from the crystal structures compared to atomistic simulations of AK [35].

**Figure 2:**
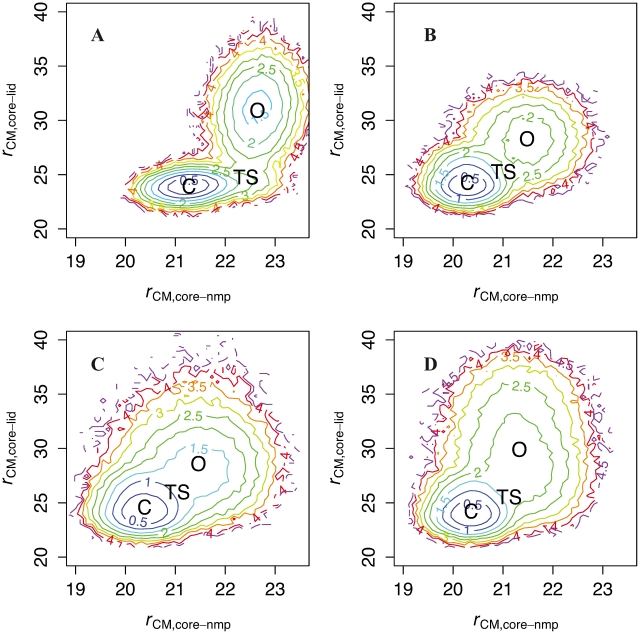
LID and NMP rigid-body motions in AKmeso, AKthermo, and variants. Potential of mean force (PMF) along *r*_CM,core-nmp_ and *r*_CM,core-lid_, which are the CORE-NMP and CORE-LID center of mass distances, respectively (units are Ångstroms). PMFs are calculated from the simulation data as described in our previous work [18]. A: AKmeso at 300K. B: AKthermo at 300K. C: AKthermo at 375K. D: AKthermo+7G at 300K. In each panel, the labels “O,” “C,” and “TS” indicate the average position of structures from each of the three respective ensembles. Contours are spaced 0.5 kcal/mol apart. All simulations are based on position-restrained minimized structures (see methods).

Figure 2 shows for key simulations the potential of mean force (PMF) describing LID and NMP quasi-rigid-body motions in Cartesian space, and Table S3 shows for each simulation the average and standard deviation of (*r*_CM,core-nmp_, *r*_CM,core-lid_) in the O ensemble. By contrast to our previous work [18], we here exclude the CORE-LID connector helices (residues 113–122 and 161–176 in AKmeso and 114–123 and 155–170 in AKthermo) from *r*_CM,core-lid_ calculations.

Panels A and B of Figure 2 compare LID and NMP closure motions in AKmeso [18] and AKthermo. Compared to AKmeso, *r*_CM,core-nmp_ is about 1 Å less in AKthermo in O, TS, and C ensembles and *r*_CM,core-lid_ is similar in C and TS ensembles, but the average *r*_CM,core-lid_ (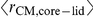) is about 2.6 Å smaller in the O ensemble. The standard deviation of *r*_CM,core-lid_, (*σ*_lid_) which we henceforth refer to as LID rigid-body flexibility, also decreases significantly in AKthermo. Thus, AKthermo has less rigid-body entropy in the O ensemble than does AKmeso. However, the two enzymes share the same basic O/C pathway; that is, LID closure is more complete in the TS than NMP closure. In addition, we previously showed that in AKmeso, the closed state is stable under apo simulation conditions (without ligand-mediated interactions in the C potential), though the O/C transition follows an NMP-first closing pathway [18] (Figure S2A). By contrast, in apo-AKthermo (Figure S2B), the fully LID-closed conformation is not stable, probably because without ligand, there are only 4 CORE-LID contacts in C, compared to 11 such contacts in AKmeso.

Heating AKthermo to 375K, at which AKthermo has a similar stability as AKmeso at 300K (see methods for details) barely increases 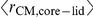 in the O ensemble (Figure 2C) ; however, *σ*_lid_ increases from 1.9 to 2.3 Å, which is comparable to AKmeso (). Thus, at domain resolution, the ensembles of AKmeso and AKthermo approximately correspond at their respective operating temperatures. Regarding mutation, AKthermo-7P (Figure S3A) produces a *r*_CM,core-lid_ distribution quite similar to that of the 375K simulation, both in 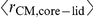 and *σ*_lid_. By contrast, Figure 2D shows that only +7G produces 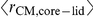 comparable to wild type AKmeso (panel A). In AKmeso, the +7P mutant does not significantly change either 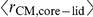 or *σ*_lid_ by more than 0.2 Å, which suggests that LID rigid-body flexibility is not determined by hinge residues in *E. coli*. Finally, AKmeso+7G (Figure S3B) expands 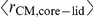 2.3 Å beyond wild type and 3.3 Å beyond AKthermo+7G, which further supports the hypothesis that hinges combine with intradomain flexibility and/or other factors to modulate the global transition. No mutant of AKmeso or AKthermo causes a change of more than 0.2 Å in either 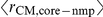 or *σ*_nmp_.

Regarding single mutations, Figure S3C shows that surprisingly, mutating only hinge 7 (P155G) does not significantly increase *σ*_lid_. The intra-LID mutant P142G+P143G affects neither 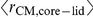 nor *σ*_lid_ by more than 0.3 Å; thus, intradomain stability of the LID does not appear to be important except for the residues preceding hinge 7. Based on observations of contact disruptions between P8 and the CORE-LID connector helices in +7G (see the later “contact probabilities” subsection for details), we also assessed P8G. Figure S3D shows that surprisingly, P8G increases *σ*_lid_ by 0.8 Å, substantially more than P155G; a slight additional enhancement occurs when P8G and P155G are combined; this suggests that the primary determinant of LID motions is *distal* to, and thus allosterically coupled to, the CORE-LID junction.

### Backbone local unfolding

For each simulation, we assess local backbone unfolding and corresponding entropy differences among O, TS, and C ensembles with the simple metric *p*_folded,*i*_ from our previous work [18], that is, the probability that pseudodihedral angle *α*_i-1,i_ is in the same rotamer (within 60°) as the native value. This metric corresponds approximately to local unfolding measured by hydrogen exchange [29]. In the thermodynamic context, regions with C-like (higher *p*_folded_, lower entropy) local unfolding in the TS ensemble will generally increase the TS-O free energy difference and thus resist O to C transitions, while O-like (lower *p*_folded_, higher entropy) regions will generally decrease the TS-C free energy difference and thus facilitate C to O transitions.

For AKmeso, we previously found that ligand-binding loop 11–13 and loop 198–199 hinder closing, while residues 42–44 in hinge 2 and 158 in hinge 7 facilitate opening [18]. As discussed in the methods, all these effects except that at hinge 7 are robust to position-restrained minimization of the wild type structures.

**Figure 3:**
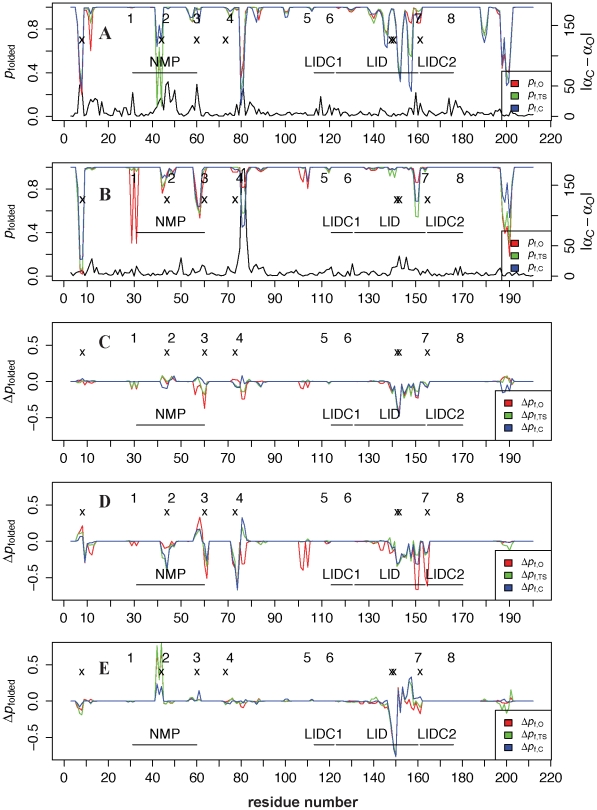
Local unfolding in open, closed, and transition state ensembles of AKmeso and AKthermo bound simulations at different temperatures. In panels A and B, *p*_folded,E_ for the pseudodihedral angle *α*_i-1,i_ is defined as fraction of structures in ensemble *E* for which *α* is in the same rotamer (within 60°) as the “reference value,” which is the average of the O and C native state values if |Δ*α*|<20°, where Δ*α* = *α*_C_−*α*_O_. To simplify the calculation for residues with |Δ*α*|≥20°, three reference values are used (O, C, and the midpoint), and the highest probability among those three is defined as *p*_folded_. The black curve shows |Δ*α*| for reference. Numbers 1–8 at the top mark the positions (respective central residues) of the eight hinges of Kern et al [14], and x's mark the 7 mutation sites. *p*_folded,TS_ is the average of the values for the closing and opening transitions. Panels A and B show AKmeso and AKthermo wild type simulations. Panels C–E show for AKthermo-7P, AKthermo+7G, and AKmeso+7P the difference in *p*_folded_ (Δ*p*_folded_) from the appropriate wild-type simulation. All simulations are at 300K.

Figure 3B shows that AKthermo is generally more folded than AKmeso (panel A) in all ensembles, especially in the LID including residues 150–151 preceding hinge 7 and loops 11–13 and hinge 2. Flexible loop 189–191 of AKmeso has no counterpart in AKthermo (see Figure 1). Surprisingly, hinges 1 and 3 are less folded in one or more ensembles in AKthermo than in AKmeso. Thus, flexibility in O and/or TS ensembles in NMP appears to have shifted away from hinge 2 in AKmeso to the N- and C-terminal hinges (1 and 3) in AKthermo, possibly because hinge 2 has been rigidified by P44.

Figure S4A shows that heating AKthermo to 375K only slightly decreases *p*_folded_ at hinge 2 and in loop 150–151 in the C ensemble. Figure 3C shows that AKthermo-7P decreases *p*_folded_ slightly at hinges 3 and 4 in the O and TS ensembles and also within LID near the mutation sites (142–143) in all ensembles, shifting partially toward AKmeso-wt. Loop 11–13 is not altered even though P8A is the only nearby sequence difference with AKmeso. Hinges 2 and 7 are relatively insensitive to the -7P mutations. Figure 3D shows that only AKthermo+7G substantially decreases *p*_folded_ in hinge 2, loop 150–151, and hinge 7; +7G also further enhances flexibility in hinge 4.

Furthermore, Figure S5C shows that in AKthermo-7P, these three hinges experience little change in 20° flexibility, which correspond approximately to order parameters, relative to AKthermo wild type Figure S5B), except for a *decrease* in such flexibility for residues following loop 42–44. While this differs with Henzler-Wildman et al. [14], who previously observed slightly enhanced simulation order parameters in these hinges; this difference may result from different length- and timescales of our CG simulations versus their atomistic simulations.

In AKmeso, relative to wild type (Figure 3A), the +7P mutant (panel E) substantially rigidifies hinge 2 and loop 156–157, which corresponds to loop 150–151 in AKthermo; ligand-binding loop 11–13 is unchanged. While the immediate vicinity of the mutations at 148–149 appears to be destabilized, the dihedral angles in this region (not shown) are sharply distributed within alternative low-energy minima in the dihedral potential and are thus more likely “refolded” than unfolded.

As expected, AKmeso+7G (Figure S4B) considerably decreases *p*_folded_ for the LID, residues 156–157 and hinge 7 in O and TS, while it surprisingly increases *p*_folded_ at hinge 2 just like +7P. This suggests that E44 in wild-type may have a low propensity for the conformation in the crystal structure compared to *either* pro or gly. In summary, the backbone dihedral results for the AKmeso and AKthermo proline and glycine mutants suggest that their sequence differences at these sites contribute partially but not fully to their differences in conformational distributions and kinetics.

Regarding single mutants, Figure S4C shows that AKthermo-P155G increases flexibility at loop 150–151 and slightly at hinge 7. P8G (panel D) only perturbs the backbone flexibility residues near the mutation site; this suggests that P8G perturbs LID rigid-body flexibility through some other, possibly allosteric mechanism. P8G+P155G shows an additive effect of these two mutants (data not shown). Interestingly, other mutants spatially close to the P8 sites also have significant functional effects. For example, glycine mutants that destabilize the CORE-LID connector helices (I116G+L168G) increase ATP binding affinity by increasing the O to C equilibrium constant [46]. In addition, forcible *in silico* unfolding of hinge 8 (proline 177) in AKmeso induces alternative contacts that stabilize C and resist the C to O transition [19].

### Contact probabilities in O, TS, and C ensembles

In our previous work, we characterized contact probabilities (*r*_ij_≤1.1 times the native contact distance) in the O, TS, and C ensembles (*p*_O_, *p*_TS_, and *p*_C_, respectively) [18]. We focused on *p*_TS_ for O-ensemble-characteristic contacts and C-ensemble-characteristic contacts, that is, those for which *p*_O_-*p*_C_≥0.2 and *p*_C_−*p*_O_≥0.2, respectively. These will be referred to henceforth simply as “O-contacts” and “C-contacts,” respectively.

**Figure 4:**
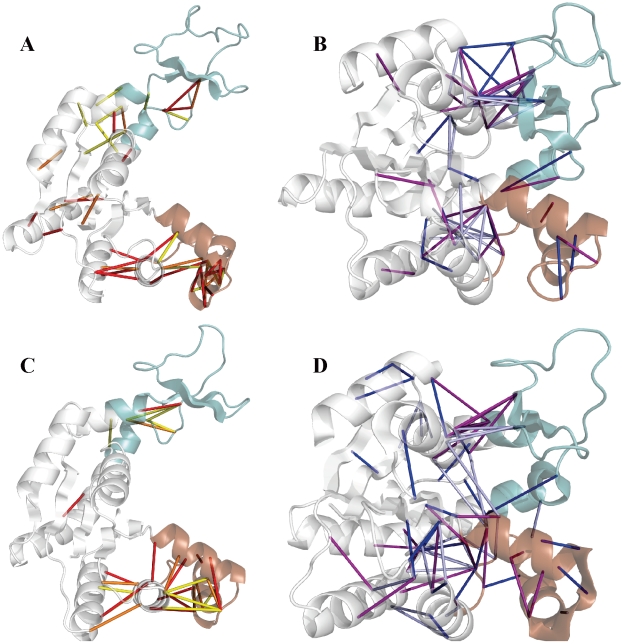
Important contacts in the transition state ensembles of the AKmeso and AKthermo bound simulations. CORE, LID and NMP domains are colored white, green, and brown, respectively. Only contacts with max(*p*_O_, *p*_C_)≥0.5 are plotted. For O-characteristic contacts (*p*_C_−*p*_O_≤−0.2), red, orange, and yellow indicate *p*_TS_≥0.7, 0.5≤*p*_TS_<0.7, and *p*_TS_<0.5, respectively. For C-characteristic contacts (*p*_C_−*p*_O_≥0.2), purple, dark blue, and light blue indicate *p*_TS_≥0.7, 0.5≤*p*_TS_<0.7, and *p*_TS_<0.5, respectively. A: O-characteristic contacts mapped onto the AKmeso O crystal structure (4AKE). B: C-characteristic contacts mapped onto the AKmeso C crystal structure (1AKE). C: O-characteristic contacts mapped onto the AKthermo O crystal structure (2RH5). D: C-characteristic contacts mapped onto the AKthermo C crystal structure (2RGX).

Figure 4A+B and C+D show the TS ensemble (TSE) contacts of AKmeso [18] and AKthermo, respectively. The TSEs of AKmeso and AKthermo share many features including the two major nuclei of C- contacts (1) at the CORE-LID interface and (2) in the lower CORE region and its interface with the NMP. In addition, consistent with LID-first closure shown in Figure 2A–B for both proteins, the CORE-LID interface is more fully formed in the TSE than the CORE-NMP interface in both proteins. Nucleus 1 includes fewer C-contacts in both TS and C in AKthermo than in AKmeso, and nucleus 2 includes more such contacts in AKthermo; most of these are in the lower CORE.

**Figure 5:**
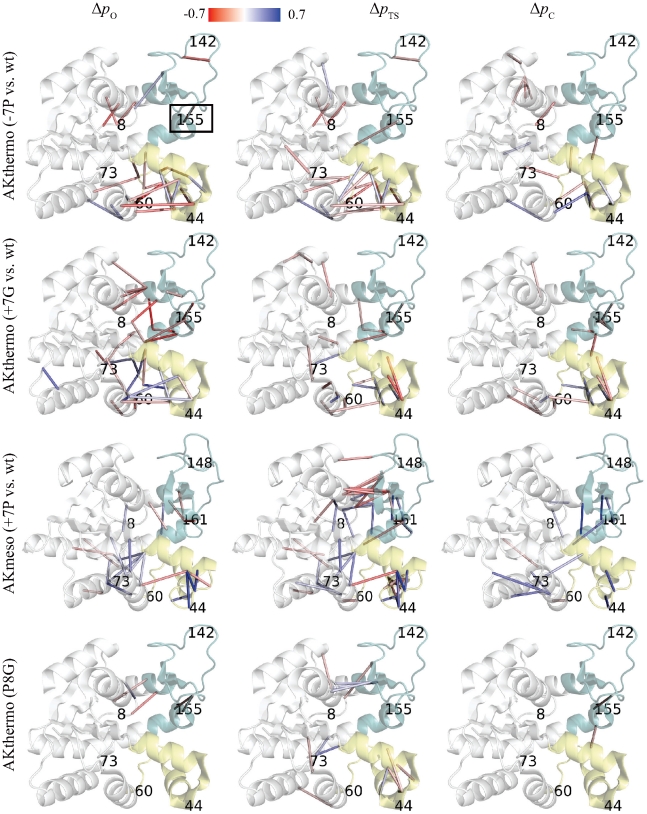
Changes in contact probabilities resulting from heating and mutations of AKthermo and AKmeso. CORE, LID and NMP domains are colored white, green, and pale yellow, respectively. Top row: AKthermo (-7P simulation – wild type simulation); Second row: AKthermo (+7G-wt); Third row: AKmeso (+7P-wt); Bottom row: AKthermo (P8G-wt). The color gradient proceeds linearly from Δ*p* = −0.7 (red) to Δ*p* = 0 (white) to Δ*p* = 0.7 (blue). Only contacts with |Δ*p*|≥0.1 are shown. The seven mutation sites are marked by their residue numbers in AKmeso or AKthermo as appropriate. These images were prepared with PyMOL. The hinge 7 (AKthermo residue 155, AKmeso residue 161) site is boxed for clarity.

Figure 5 and Figure S6 reveal the effects of heating and proline and glycine mutants upon the AKthermo and AKmeso contact networks by comparing *p*_O_, *p*_TS,_ and *p*_C_ between the wild type and perturbed simulations for all contacts that are O- or C-characteristic in either simulation. Figure S6, top row shows that heating AKthermo from 300K to 375K produces only small effects on a minority of the contacts shown in Figure 4C+D, and these are distributed broadly around the CORE-LID and CORE-NMP interface regions.

Figure 5, top row shows that the -7P mutant locally destabilizes the contact network of AKthermo. As with heated conditions, little changes near nucleus 1 in O (the CORE-LID interface region), except some contacts between the residue 8 region and the CORE-LID connector helices in O and TS and a single contact near hinge 7 in O and TS. By contrast, at nucleus 2 (lower CORE and its interface with NMP), multiple contacts are destabilized in O and TS; this may reflect a shift toward the comparatively weaker nucleus 2 seen in wild type AKmeso. In addition, -7P produces more and stronger contact perturbations than heating to 375K, which suggests that the mutant has a larger “enthalpic” effect than heating. The second row shows that +7G disrupts nucleus 1 much more than -7P, which is probably linked to the greater increase in LID rigid-body flexibility in +7G than in -7P.

The third row shows that relative to wild-type AKmeso, the +7P mutant generally stabilizes the contact network, which is opposite to the effect of AKthermo-7P. Consistent with the rigidification of hinge 2 by +7P (Figure 3E), contacts near hinge 2 (position 44) strengthen substantially in O and TS. In addition, the CORE-LID interface peculiarly weakens in the TS, though result is suspect since as reported in the methods, the same contacts are artifactually strengthened by wild-type minimization. Some lower CORE contacts are also stabilized in O and TS. Figure S6, second row shows that as expected, AKmeso+7G generally destabilizes the contact network, with the exception of contacts around hinge 2, which is consistent with the surprising backbone rigidification there (Figure S4B).

Regarding AKthermo single-site mutants, P155G (Figure S6, bottom row) alters the contact network mainly near G155, and P8G (Figure 5, bottom row) primarily alters the contacts between CORE and CORE-LID connector helix 1. Given that P8G accounts for most of the enhancement of LID rigid-body flexibility seen in AKthermo+7G (Figure S3D), the contacts between CORE and CORE-LID connector helix 1 are likely the most important for modulating the global transition. Combining P8G and P155G (not shown) produces an approximately additive effect, perturbing nucleus 1 in a similar way as +7G (Figure 5, second row).

## Discussion

### What differentiates AKmeso and AKthermo?

Mesophile/thermophile homologous pairs of enzymes provide useful model systems to investigate the sequence and structural basis for functionally important motions. In ideal cases, the ensembles of the two enzymes correspond at their active temperatures [21], [22]. While there is ample evidence that AKthermo is both more stable [26], [29] and less flexible [3], [14] at room temperature than AKmeso, the precise locations and mechanisms of critical residues and regions remain poorly understood. Here, within the framework of a coarse-grained model, we compared many different types of motions between AKmeso and AKthermo and identified differences in critical residues and contacts. Combined with the computational proline and glycine mutants, our results suggest that both backbone dihedral flexibility and global functional motions are correlated with, but not exclusively determined by, the intrinsic flexibility of the hinge residues, whether pro, the AKmeso residue, or gly.

Regarding the global structural transition, AKmeso and AKthermo are more similar than different. For example, in both enzymes, LID prefers to close before NMP in the presence of ligand (Figure 2A–B). These results support the hypothesis that global mechanistic properties depend more upon the topology of structure than on local variations in sequence [38]–[40].

Inferring the impact of the observed motional differences between AKmeso and AKthermo upon the kinetic properties at room temperature is more challenging. For any given degree of freedom, its impact on kinetics will depend on whether it is more similar to O or C in the TS. Such impacts are most easily assessed by comparing wild type and mutant simulations because of the much higher level of sequence identity between the two than between AKmeso and AKthermo. Given that C is generally the least flexible state with more contacts, perturbation of a degree of freedom that is more like C in the TS will change the free energy of O while leaving Δ*G*_TS-C_ unchanged, thus primarily affecting Δ*G*_TS-O_ and hence the closing rate (Figure S7A). Perturbation of a degree of freedom that is O-like in the TS will shift the free energies of O and TS in concert relative to C, thus primarily affecting Δ*G*_TS-C_ and thus the opening rate (Figure S7B).

For example, perturbation of LID rigid-body flexibility will primarily affect the LID-closing rate since this degree of freedom is C-like (rigid, low-entropy) in the TS in AKmeso, AKthermo, and all the mutants. Specifically, decreasing (increasing) this flexibility in O will make Δ*S*_TS-O_ less (more) negative, thus facilitating (resisting) closing. Thus, since AKmeso and AKthermo+7G have higher LID rigid-body flexibility than wild-type AKthermo (Figure 2), we predict that AKmeso and AKthermo+7G will have a slower closing rate. Similarly, perturbing the foldedness of hinge 2, which is O-like (flexible) in the TS of AKmeso, will primarily affect the opening rate. Since wild-type AKthermo and AKmeso+7P are less flexible in O and TS at hinge 2 than wild-type AKmeso (Figure 3), they will have a less positive Δ*S*_TS-C_ and thus a slower NMP-opening rate than wild-type AKmeso. LID foldedness is substantially higher in AKthermo than in AKmeso (Figure 3); however, this difference between the two proteins is relatively uniform in all ensembles and is not predicted to affect O/C kinetics.

However, these predicted kinetic effects in Cartesian and dihedral space may be compensated by quasi-enthalpic changes in contacts. For example, Figure 5, row 2 shows that in AKthermo+7G, a cluster of contacts in the CORE-LID connector and interface region is destabilized in O but not C or TS, which will make Δ*H*_TS-O_ more negative and thus facilitate closing, compensating for the more negative Δ*S*_TS-O_ due to increased LID rigid-body flexibility in O. Similarly, in AKmeso+7P, (Figure 5, third row), contacts near hinge 2 are substantially stabilized in both O and TS but not C, which will make Δ*H*_TS-C_ less positive and accelerate NMP opening, which counters the resistive effect of reduced hinge 2 flexibility on *k*_O_. Thus, most motional perturbations involve enthalpy/entropy compensation, and prediction of which of these effects (ΔΔ*H* or *T*ΔΔ*S*) will be greater, and thus the net effect on *k*_O_ or *k*_C_, is beyond the scope of a coarse-grained model. However, given that heating and some mutations are predicted to partially or fully interconvert some of the key entropic and enthalpic features of the two enzymes, it is plausible that a significant fraction of these sites will turn out to be important for O/C kinetic differences between the enzymes.

The prediction that perturbations at the CORE-LID interface and junction will primarily affect the rate of LID *closing* differs with the hypothesis of Wolf-Watz et al. [3] that the LID-opening rate differs more between AKmeso and AKthermo than does the closing rate. However, the LID-first closing on which our prediction depends has been seen in prior AK calculations and simulations [31], [35], [47].

In a different AKmeso/thermo pair with 74% sequence identity (from the *Bacillus* genus), Bae et al. showed that swapping the entire LID and NMP domains between the two enzymes interconverts catalytic properties, but swapping the CORE-LID hinges does not [26]. However, since for these two AKs, the CORE-LID hinge sequences are very similar, including a glutamate (E164) at the position equivalent to *Aquifex* P155 and *E. coli* E161, it is difficult to infer from this work a negative result about the role of hinges in AKs in general. We did not simulate the *Bacillus* AK conformational transitions in this work since only the closed structures [48] are available. Thus, our mutational results complement prior works in the field [14], [26] in suggesting that prolines and loop length reduction can promote restricted global dynamics in proteins but are not an exclusive mechanism for doing so or an essential property of thermophilic AKs. In addition, more drastic intradomain perturbations, for example unfolding LID by mutating some residues to glycine, can dramatically affect the relative populations of O and C [49]; however, the kinetic effect of such a perturbation is less clear.

### The corresponding states hypothesis in AK

Figure 2 and Table S3 show that LID rigid-body flexibility in AKmeso at 300K (σ_lid_) is closer to AKthermo at 375K than to AKthermo at 300K. Thus, at the domain motion scale, adenylate kinase approximately follows the corresponding states hypothesis [20]. At residue and contact motional scales, heating will necessarily increase local unfolding and weaken the contact network. For some dihedrals that are less stable in AKmeso than AKthermo (hinge 2 and loop 150–151 preceding hinge 7), small decreases of *p*_folded_ in O and/or TS toward AKmeso occur (Figure S4A). However, these are not as large as the backbone flexibility changes induced by the -7P mutant, even though these two conditions produce similar effects on LID rigid-body flexibility; this suggests that heating modulates global dynamics through a more distributed mechanism. Thus, our results suggest that the AKmeso and AKthermo ensembles approximately correspond at their optimal temperatures, to the extent thermodynamically possible. This is consistent with the local, but not global, correspondence of ensembles between psychrophilic and thermophilic alcohol dehydrogenases at their respective operating temperatures [23].

### Applications to design of flexibility and allostery

Our results also have general applications to the design of allostery and flexibility in proteins, especially in regard to the hypothesis that evolution selects certain amino acids (e.g. proline, glycine) or sequences to calibrate the flexibility in key regions to the operating temperature of the organism and other constraints. Our results generally support the protein engineering hypothesis that proline removal (e.g. in AKthermo) and/or glycine insertion loosen functionally important flexibility, while proline insertion (e.g. in AKmeso) represses such flexibility, expanding on the hypothesis of Henzler-Wildman et al. [14].

The likely importance of flexibility outside the hinge regions, including for example the apparent allosteric effect of position 8 distal to the CORE-LID junction, certainly presents a challenge for engineering allosteric motions. While swapping entire domains as done by Bae et al. [26] is not likely to be effective computationally due to the severity of structural perturbation involved, targeting other rigid residues within LID and/or NMP with glycine or alanine mutation might effectively perturb allosterically important intradomain stability. Indeed, multiple mutations in a local region of sequence and/or structure are required to increase the stabilities of mesophilic adenylate kinases [50], [51]. Mutation to glycine or alanine may be most effective at positions that have sampled these amino acids in multiple sequence alignments. For example, only one of the four “purely rational” helix-capping mutations attempted by Speurgin et al. stabilized the helix without disrupting stability and/or activity [52]. Furthermore, steric repulsion and/or side-chain interactions may also be important.

Local structural entropy (LSE) quantifies the propensity of local sequence fragments to populate different secondary structures [53]. Rational perturbation of LSE has successfully stabilized *Bacillus* AKmeso [50], and it is conceivable that LSE perturbations could also change functionally important flexibility. For example, Figure S8 shows that for residues 148–53, which immediately follow the 141–147 insertion in LID (*E. coli* numbers), LSE is substantially lower in AKthermo than in AKmeso. Thus, swapping AKthermo residues into AKmeso in this region may increase the coupling between hinge 7 flexibility and LID rigid-body flexibility and reduce LID rigid-body flexibility where the -7P mutant failed.

In addition to LSE, the evolutionary trace via PSI-BLAST [54] could suggest additional productive mutations. Some of these may be counterintuitive; for example, gly insertion from a homologous thermophile into mesophilic RNAse H *stabilizes* an alternate conformer, making the conformational exchange slower like in the thermophilic enzyme [55].

### Limitations

The use of a coarse-grained model for this problem has both limitations and advantages. For example, the minimization and simulation protocol cannot capture perturbations in the average (free energy minimum) structure that may result from mutations. On the other hand, in addition to computational efficiency and lack of bias in the potential, the CG model *can* in principle isolate the dynamic/entropic effect of a mutation to a more flexible (e.g. gly) or less flexible (e.g. pro) residue from its effect on the average structure. That is, it can measure the propagation of a local flexibility change through structure at low resolution and possibly stimulate focused atomistic simulations and experiments. In this model, we might further refine our conclusions by atomistic simulations of key variants, especially for mutants like AKthermo+7G and AKmeso+7P that are expected to greatly alter local and/or global dynamics.

Dihedral predictions likely have approximately residue resolution since the CG α angles lie in the same plane as the underlying ϕ and ψ angles; contact predictions may be accurate at the level of clusters of contacts but not for individual ones, since the CG contacts are necessarily measured between C_α_ atoms rather than between residue or side-chain centroids. Indeed, mutants with the largest effects on global dynamics (e.g. AKthermo+7G) often affect large clusters of contacts.

Another variable that can affect these simulations and other works are the different environments under which calculations and/or measurements are made. *In silico*, we calibrate our simulation so that the O and C wells are within 2 Å C_α_ rmsd of the crystal structures. By contrast, protein dynamic experiments like native state hydrogen/deuterium exchange (HX) and NMR relaxation are performed in dilute solution conditions, and HX measures unfolding reactions which may occur on timescales different from the ms^-1^ timescale of the AK O/C transition. *In vivo*, crowding from other biomolecules shifts the conformational distribution, especially by stabilizing C relative to O [56] and possibly repressing the domain-scale unfolding that has been observed by HX for AKthermo and especially AKmeso under ligand-free conditions [29]. Thus, any given simulation or experiment only approximates reality, and further iteration among different kinds of simulations and experiments will refine the understanding in this area toward the most functionally relevant predictions.

### Conclusions

This study characterizes conformational ensembles in detail in two allosteric-like proteins in which flexibility and catalysis are intimately linked. While AKmeso and AKthermo share a LID-first closure pathway in the presence of ligand, LID rigid-body flexibility is considerably less in the O ensemble of AKthermo than in that of AKmeso. While AKthermo is generally more locally folded than AKmeso, important backbone dihedrals have shifted to nearby locations in some regions of the protein. Consistent with the corresponding states hypothesis, heating of AKthermo increases LID rigid-body flexibility in the O ensemble. The AKthermo and AKmeso proline and glycine mutants demonstrate that hinge sites modulate functional motions in AK but do not exclusively account for AKmeso/AKthermo differences, possibly due to contributions from intradomain flexibility. Overall, the results of this coarse-grained simulation, analysis, and design approach suggest that this approach is a generally effective first step toward (re)design of flexibility and allostery in proteins.

## Methods

### Double-well Gō model setup and simulation conditions

For each of two (O and C) conformations, the algorithm of Karanicolas & Brooks [57], [58] creates a single-well Gō potential with the aid of the MMTSB server (www.mmtsb.org). We combine the potentials for open and closed structures using the exponential averaging approach and generic pseudo-bond angle potential of Best and Hummer [37]. This approach creates a smooth double-well potential for which relative stability of the two states and the transition frequency between them can be calibrated. Simulations are carried out with the molecular dynamics program CHARMM [59], [60] for 750 ns (50 million 15-fs time steps) per simulation. Based on our previous observation that multiple independent 750-ns simulations of wild-type AKmeso produced nearly identical results in dihedral and contact space [18], we infer that a single 750 ns simulation per variant in this work is adequate for convergence. Langevin dynamics are carried out as in the double-well Gō model of Best and Hummer [37].

We add ligand-mediated interactions to the C potentials for the bound AKthermo simulations using the same cutoffs, criteria and other parameters as for AKmeso [18]; this results in a very similar number of interactions (21 vs. 22 in AKmeso) with a similar structural distribution (see Figure S9). For mutants of both AKthermo and AKmeso, we used the ligand-mediated interaction set from wild type to avoid any distortion artifacts arising from the minimization of side chains in mutant construction. This is reasonable since none of the mutated residues contacts the ligand in either protein.

Previously, we scaled the contact energies by a factor of 2.5 in O and C potentials [18] to compensate for extra conformational entropy induced by the generic bond angle potential [37]. As for prior double-well Gō models of other proteins [37], [61], we chose the scale factor to achieve *Q*_C,all_ of approximately 0.9 in the bound form, where *Q*_C,all_ is the fraction of all closed state contacts (including those shared with O). This also resulted in about 2.0 Å *rms*_C_ (C_α_ rmsd with respect to the closed crystal structure) for the C native basin, which is approximately the *rms*_C_ attained in prior atomistic simulations of AK [35]. In AKthermo, we scale the contacts by the same absolute factor of 2.5 so that equivalent pairs of interacting residues have identical energies in AKmeso and AKthermo; this results in *Q*_C,all_∼0.94 and *rms*_C_∼1.5 Å in the C well at 300K. To assess the effect of this scaling, we briefly compare these AKthermo conditions to a “weak” simulation that does not correct the raw wild-type AKthermo scale to that of AKmeso. Table S3 shows that in Cartesian space, the weak simulation negligibly changes 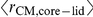 and σ_lid_. In all ensembles, *p*_folded_ differs by less than 0.1 with the regular (“strong”) simulation, and there are few contacts with any change in probability in any of the three ensembles (not shown).

To approximately test the corresponding states hypothesis [20] for AKthermo, we identify from single-well simulations a simulation temperature at which the stability of AKthermo is similar to the targeted stability of *Q*_C,all_ = 0.90 for AKmeso at 300K that have formed in single-well simulations. These simulations (not shown) show that AKthermo attains *Q*_C,all_∼0.90 at 375K, which we therefore use for the high-temperature simulations of AKthermo.

From the opening and closing rates experimentally measured by Wolf-Watz et al. [3], we estimate *K*_eq_ = *k*_close_/*k*_open_ = 1571s^−1^/44s^−1^ = 35.7 in favor of C for AKthermo, compared with 1374/286 = 4.8 for AKmeso. We simulate wild-type AKthermo calibrated to both *K*_eq_ = 4.5 and *K*_eq_ = 16.3 (the closest we could attain to 35.7), and differences between these two simulations are limited (not shown). The average positions of O, TS, and C ensembles in (*r*_CM,core-nmp_; *r*_CM,core-lid_) space are essentially identical. Hinge 2 decreases slightly (<0.1) in *p*_folded_ in O and TS. There are very few contacts with any change in probability in any of the three ensembles relative to the *K*_eq_ = 4.5 simulation. Due to the insensitivity to the calibrated *K*_eq_, we use the *K*_eq_ = 4.5 AKthermo wild type simulation for most results in this work since it samples both O and C ensembles adequately. For a given simulation, we calculate *K*_eq_ as the ratio between the number of structures in the closed and open basins.

To identify kinetic properties from a given simulation, we partition the trajectory structures into O, C, and TS ensembles as in our published wild-type AKmeso simulations [18] by identifying the separatrix line in (*Q*_O_, *Q*_C_) space that meets the criterion previously used by Hummer et al. [44], [45] for one-dimensional reaction coordinates. As opposed to *Q*_O(C),all_, *Q*_O(C)_ indicates contacts unique to the O(C) crystal structure. According to the Hummer criterion [44], [45], the separatrix will have optimal *p*_TP_, that is, maximal selectivity for structures that lie on lie along “reactive” transition paths that cross between the O and C wells. Since for AKthermo the O and C wells in AKthermo are not as well-separated along *Q*_O_ as they are in AKmeso (Figure S10B vs. Figure S10A), we identify the O well by the first maximum in the distribution of *Q*_C_ rather than by the second maximum in the distribution of *Q*_O_ as before [18]. Table S1 shows that for the simulations in this work, values of *p*_TP_ are typically between 0.35 and 0.45 when O- and C-characteristic contact sets are optimized [18], compared to a theoretical maximum of 0.5 [44].

### Mutant construction and minimization

We initially constructed mutants using the mutagenesis wizard of PyMOL. For applicable AKthermo (AKmeso) mutant residues, we selected the rotamer of the equivalent residue in the AKmeso (AKthermo) structure when possible. In our AKmeso paper, we based the double-well Gō potential upon the contacts in the O and C crystal structures [18]. While this protocol can also be applied to the wild-type AKthermo simulation, the raw mutant structures from PyMOL may contain steric clashes and other features that are unsuitable for creating the Gō potentials. To address this problem, we seek to develop an initial conformation preparation protocol that permits relief of local strain caused by the mutants but prevents extraneous relaxation at distal positions. For such a protocol, we establish the goal that simulations under Gō potentials derived from minimized structures of the wild-type crystal structures should deviate minimally from simulations based on the crystal structures themselves. We carry out all minimization of mutants using GROMACS 4.5 [62] with the CHARMM22 force field including the CMAP backbone dihedral correction [63] for 5000 steps to remove steric clashes. In C state potentials, we include the ATP and AMP atoms with CHARMM parameters determined by the SwissParam server [64], but not the bridging phosphates. For each mutant, these minimized O and C structures were used to determine the Gō contact potentials for O and C.

First, we test the effect of unrestrained minimization of the wild-type initial conformations. Table S3 shows that relative to the crystal structure-based simulations (M-xtal and T-xtal), this protocol decreases the average *r*_CM,core-lid_ by 2.3 Å in AKmeso and increases it by 0.7 Å in AKthermo. To correct for these probably artifactual results, we tested initial conformations of wild type for which all residues but the 7 mutant sites and atoms from near neighbors are position-restrained with force constants of 1000 kJ/mol/nm^2^ in x,y, and z directions. We define near neighbors as residues with 10 or more 4.5 Å atomic contacts to a mutant site in the wild type or the appropriate 7-site mutant (−7P, +7G, or +7P) structure; the non-restrained residues comprise 12–15% of the protein. By contrast to unrestrained minimization, such position-restrained minimization (M-wt and T-wt) shifts the average *r*_CM,core-lid_ by less than 0.5 Å in either AKmeso or AKthermo. Thus, the Gō simulation results are surprisingly sensitive to minor variations in the starting conformation. This may result from the pairwise nature of contact interactions, which can be perturbed by the motion of either residue, especially in models like the Karanicolas model [57] that use a discrete contact distance cutoff.

Figure S11 shows that for AKthermo, neither unrestrained (B) nor restrained (C) minimization greatly perturbs the dihedral flexibility relative to the crystal structure-based simulation (panel A). Both reduce *p*_folded_ by about 0.2 for residues 150–151, the loop immediately preceding hinge 7, and loop 187–191, though restrained minimization reduces *p*_folded,C_ less at hinge 3. By contrast, *p*_folded_ for the O ensemble in loop 150–151 is about 0.6 less in +7G mutant than in wild type with position-restrained minimization. By contrast for AKmeso, Figure S12 shows that unrestrained minimization (B) reduces *p*_folded,C_ at hinge 3 by ∼0.3 and for residues 86–87 by ∼0.6 and increases *p*_folded,TS_ by ∼0.4 for hinge 2; restrained minimization produces *p*_folded_ much closer to wild type at all these positions. Both kinds of minimization stabilize local folding at hinge 7, suggesting the previously observed behavior for AKmeso at hinge 7 [18] is not a robust feature, unlike that near hinge 2. This is consistent with the observation that in AKthermo, LID rigid-body flexibility (which is robust to restrained minimization) is increased more by the P8G mutation (Figure S3D) than by the P155G mutation at hinge 7 (Figure S3C).

Based on these Cartesian and dihedral results, we employ the position restrained minimization approach to all wild type, heated, and mutant simulations presented in this work. Figure S13 shows that for both AKmeso and AKthermo, while a number of contacts increase or decrease in probability, most of these changes are compensated by nearby contacts with opposite changes. The exception is a cluster of strengthened O- and TS-contacts surrounding position 161 (hinge 7) in AKmeso; this is correlated with the increase in stability of hinge 7 seen upon minimization (Figure S12C). We do not consider these contact changes in our decision since these will be overly sensitive to distinguish between minimization protocols due to their pairwise nature as described above.

## Supporting Information

Figure S1 **Structural differences between wild-type AKmeso and AKthermo in O and C states.** A: Superposition of the AKthermo open structure (2RH5 [42], orange) on the AKmeso open structure (4AKE [65], blue) based on equivalent positions in the CORE domain. B: Corresponding superposition of the AKthermo closed structure (2RGX [42], orange) on the AKmeso closed structure (1AKE [66], blue). In (B) and (C), arrows indicate the locations of the two CORE-LID connector helices.
(PDF)

Figure S2 **LID and NMP rigid-body motions in AKmeso and AKthermo apo simulations.** PMFs are calculated from apo simulations of AKmeso (A) and AKthermo (B) and are labeled as described in the legend of Figure 2 the main text.
(PDF)

Figure S3 **LID and NMP rigid-body motions in additional variants of AKmeso and AKthermo.** A: AKthermo-7P; B: AKmeso+7G; C: AKthermo P155G; D: AKthermo + P8G. PMFs are labeled as described in the legend of Figure 2 the main text.
(PDF)

Figure S4 **Local unfolding in additional variants of AKmeso and AKthermo.** A: AKmeso at 375K. B: AKmeso+7G at 300K; C: AKthermo P155G at 300K; D: AKthermo P8G at 300K. Panels are labeled as in Figure 3 of the main text.
(PDF)

Figure S5 **Small-scale backbone flexibility in key simulations.** In each panel, *p*_rigid,E_ for the pseudodihedral angle *α*_i-1,i_ is defined as fraction of structures in ensemble *E* for which *α* is in the same rotamer (within 20°) as the “reference value,” which is the average of the O and C native state values if |Δ*α*|<20°, where Δ*α* = *α*_C_−*α*_O_. To simplify the calculation for residues with |Δ*α*|≥20°, three reference values are used (O, C, and the midpoint), and the highest probability among those three is defined as *p*_rigid_. The black curve shows |Δ*α*| for reference. Numbers 1–8 at the top mark the positions (respective central residues) of the eight hinges of Kern et al [14], and x's mark the 7 mutation sites. *p*_rigid,TS_ is the average of the values for the closing and opening transitions. A: AKmeso; B: AKthermo. Panels C and D show the difference in *p*_rigid_ (Δ*p*_rigid_) from the appropriate wild-type simulation for AKthermo-7P and AKmeso+7P, respectively.
(PDF)

Figure S6 **Changes in contact probabilities resulting from additional variants of AKthermo.** Top row: AKthermo (375K-300K simulation); Second row: AKmeso (+7G-wt); Third row: AKthermo (P155G-wt). Otherwise, panels are labeled and colored as in Figure 5 of the main text.
(PDF)

Figure S7 **Thermodynamic and kinetic effects of perturbing different types of degrees of freedom.** A: For a degree of freedom that is C-like in the TS, perturbations will primarily affect the free energy of O, and thus the TS-O free energy difference and the closing rate. B: For a degree of freedom that is O-like in the TS, perturbation will affect the free energies of O and TS similarly, modulating the TS-C free energy and by extension the opening rate.
(PDF)

Figure S8 **Differences in local structural entropy (LSE) between AKmeso and AKthermo.** Red indicates residues for which LSE is lower by 0.15 or more in AKthermo; blue indicates that LSE is lower by 0.15 or more in AKmeso. Brown sticks indicate the substrate analog.
(PNG)

Figure S9 **Ligand-mediated interactions mapped onto the closed** ***Aquifex*** **AK crystal structure (2RGX).** CORE, LID, and NMP domains are shown in green, blue, and red, respectively. Ligand-mediated interactions are indicated by pseudo-bonds between the C_α_ atoms of involved residues.
(PNG)

Figure S10 **The bound AKmeso and AKthermo transitions in global contact reaction coordinates.** Estimated potential of mean force (PMF) for AKmeso (A) and AKthermo (B) simulations based on projection of simulation data onto *Q*_O_ and *Q*_C_ reaction coordinates, where *Q*_O(C)_ is the fraction of native contacts unique to the O(C) crystal structure. The diagonal lines show the transition separatrices calculated as in our previous work. Contours are spaced at 0.5 kcal/mol intervals in both PMFs.
(PDF)

Figure S11 **Local unfolding in different minimization variants of AKthermo.** A: unminimized simulation; B and C: for unrestrained minimization and position-restrained minimization, respectively, Δ*p*_folded_ vs. the unminimized simulation. Panels are labeled as in Figure 3 of the main text.
(PDF)

Figure S12 **Local unfolding in different minimization variants of AKmeso.** A: unminimized simulation; B and C: for unrestrained minimization and position-restrained minimization, respectively, Δ*p*_folded_ vs. the unminimized simulation. Panels are labeled as in Figure 3 of the main text.
(PDF)

Figure S13 **Changes in contact probabilities between crystal structure-based wild type simulations and wild-type simulations after position-restrained minimization.** Panels are labeled and colored as in Figure 5 of the main text.
(PDF)

Table S1 **Double-well Gō model parameters and statistics for simulations in this work.**
(DOC)

Table S2 **Positions of O and C wells for simulations in this work.**
(DOC)

Table S3 **Average (** ***r*** **_CM,core-nmp_,** ***r*** **_CM,core-lid_) positions and standard deviations of the O ensemble for simulations in this work.**
(DOC)
