# Supplementary figures and images for: Interconversion of Functional Motions between Mesophilic and Thermophilic Adenylate Kinases
Source: PLoS Comput Biol. 2011 Jul 14;7(7):e1002103. doi: 10.1371/journal.pcbi.1002103 (PMC3136430; doi:10.1371/journal.pcbi.1002103)

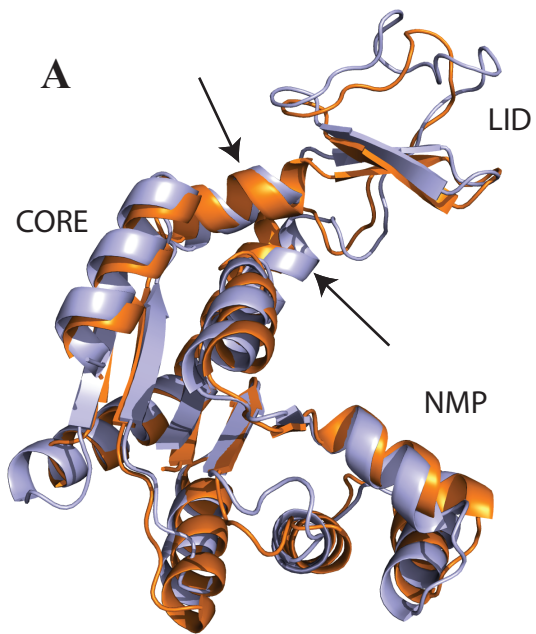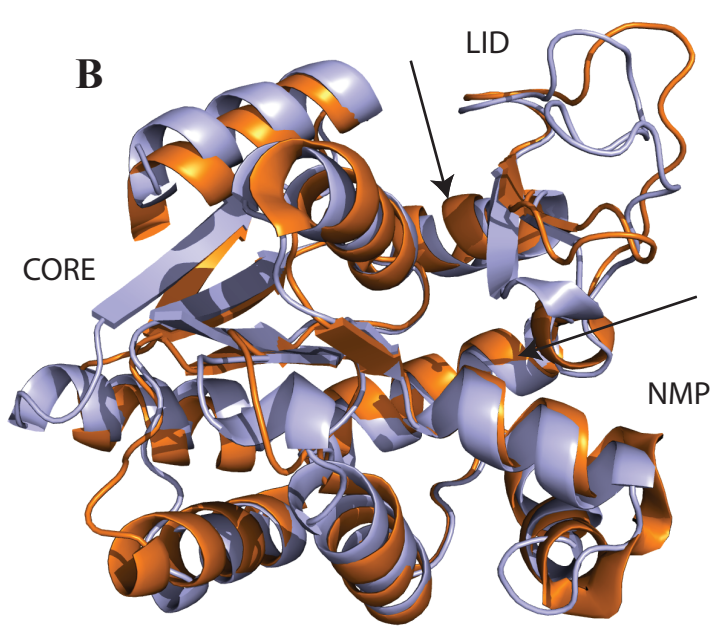

Supplement: Figure S1 — Structural differences between wild-type AKmeso and AKthermo in O and C states. A: Superposition of the AKthermo open structure (2RH5 [42], orange) on the AKmeso open structure (4AKE [65], blue) based on equivalent positions in the CORE domain. B: Corresponding superposition of the AKthermo closed structure (2RGX [42], orange) on the AKmeso closed structure (1AKE [66], blue). In (B) and (C), arrows indicate the locations of the two CORE-LID connector helices. (PDF) [file pcbi.1002103.s001.pdf]

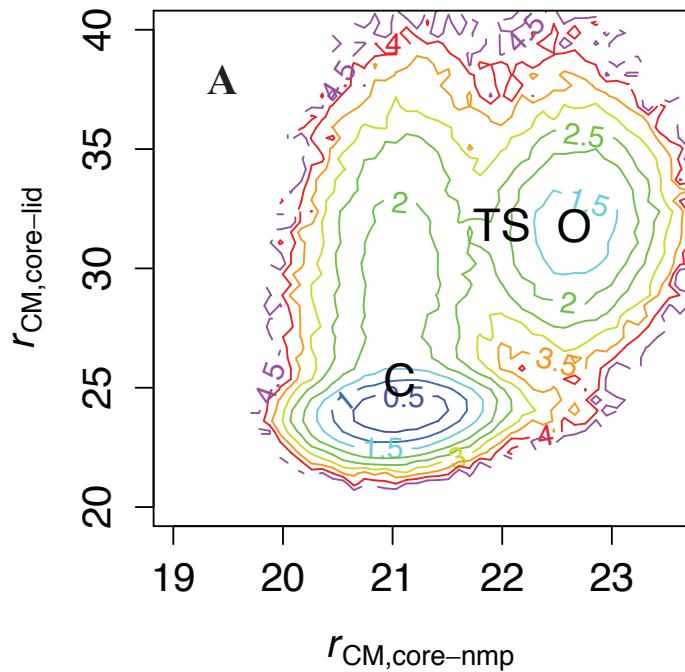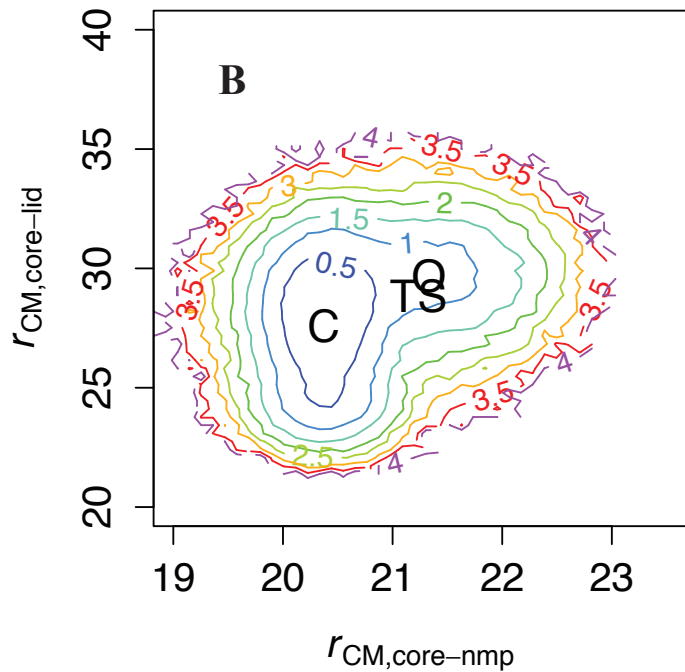

Supplement: Figure S2 — LID and NMP rigid-body motions in AKmeso and AKthermo apo simulations. PMFs are calculated from apo simulations of AKmeso (A) and AKthermo (B) and are labeled as described in the legend of Figure 2 the main text. (PDF) [file pcbi.1002103.s002.pdf]

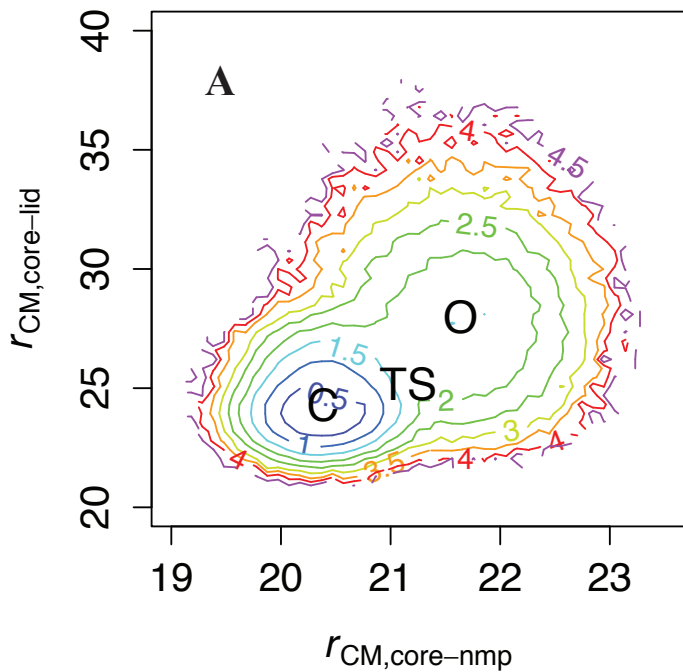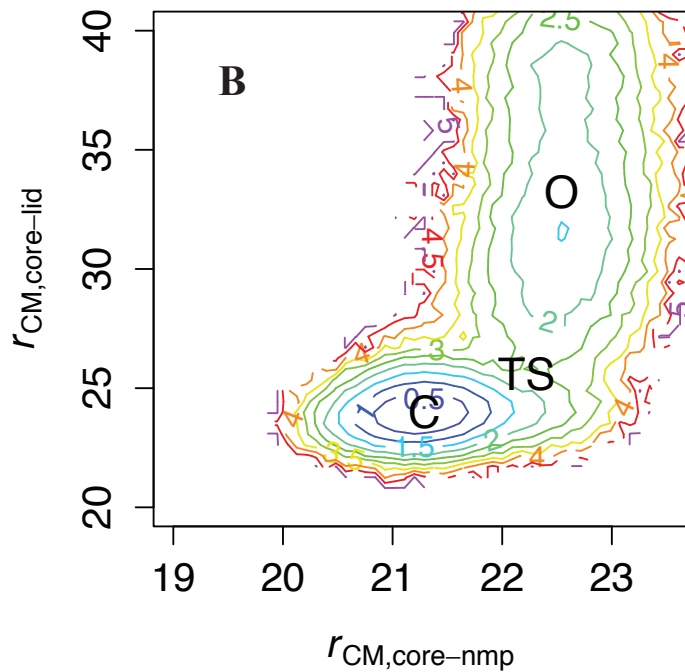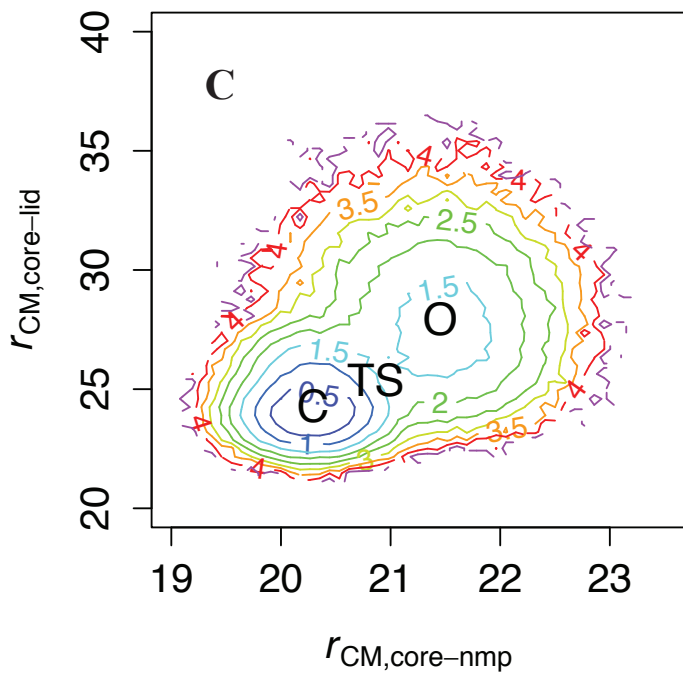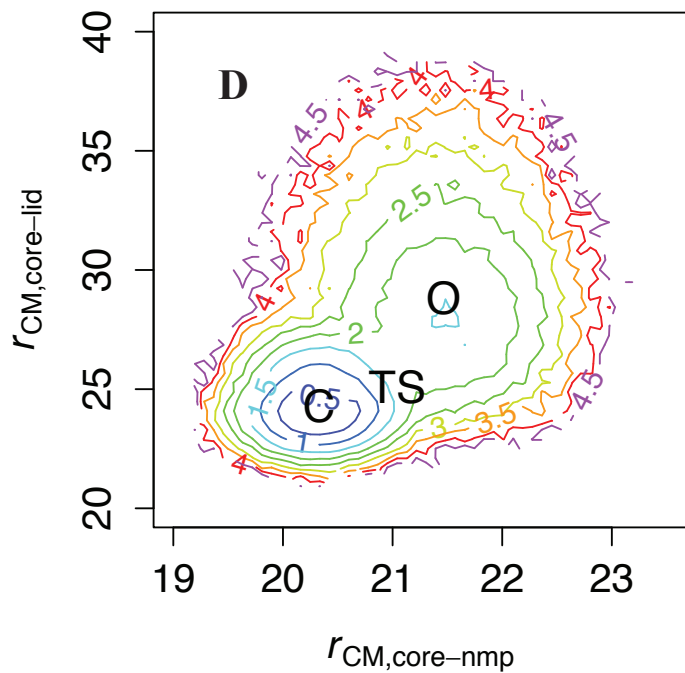

Supplement: Figure S3 — LID and NMP rigid-body motions in additional variants of AKmeso and AKthermo. A: AKthermo-7P; B: AKmeso+7G; C: AKthermo P155G; D: AKthermo + P8G. PMFs are labeled as described in the legend of Figure 2 the main text. (PDF) [file pcbi.1002103.s003.pdf]

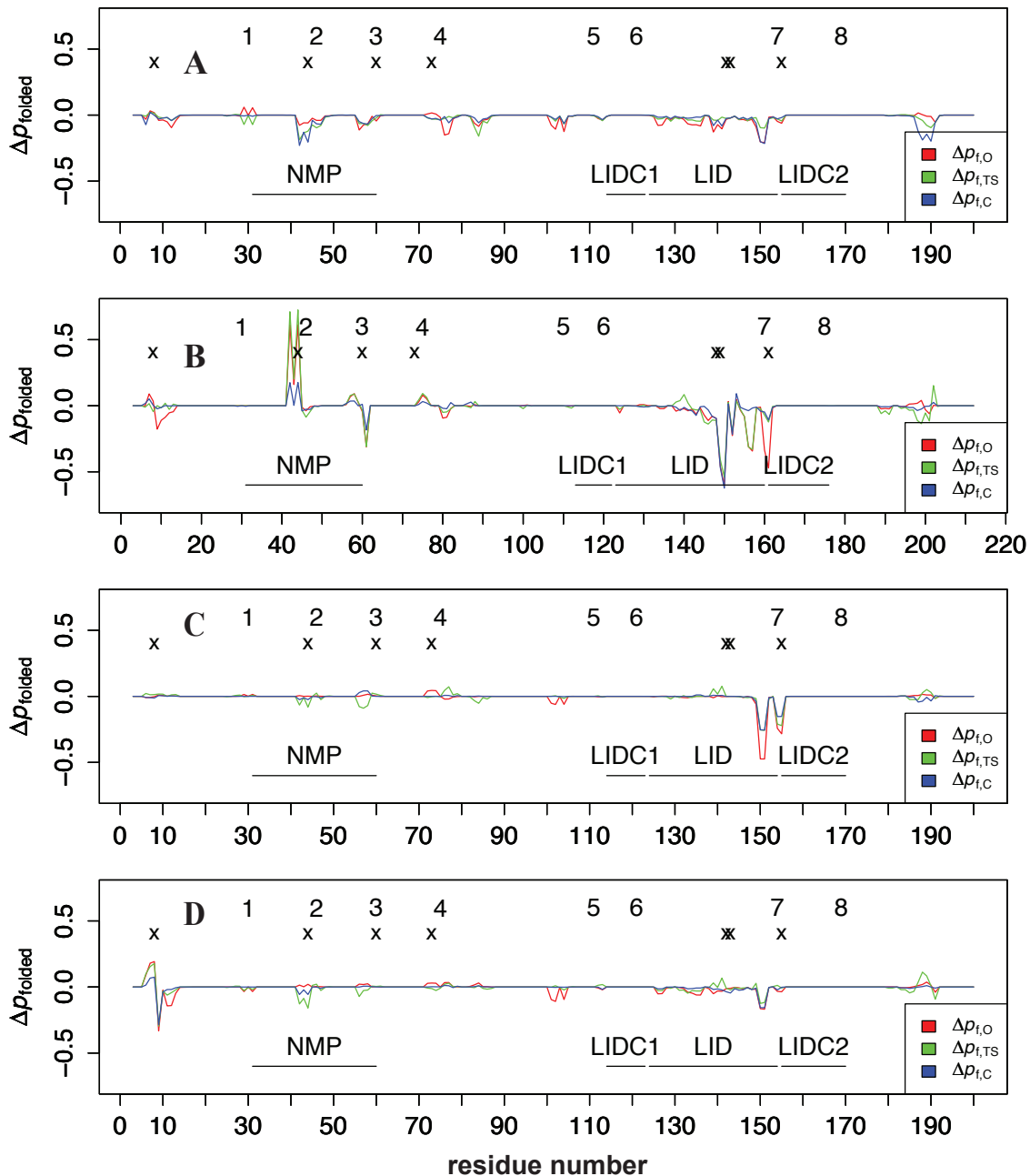

Supplement: Figure S4 — Local unfolding in additional variants of AKmeso and AKthermo. A: AKmeso at 375K. B: AKmeso+7G at 300K; C: AKthermo P155G at 300K; D: AKthermo P8G at 300K. Panels are labeled as in Figure 3 of the main text. (PDF) [file pcbi.1002103.s004.pdf]

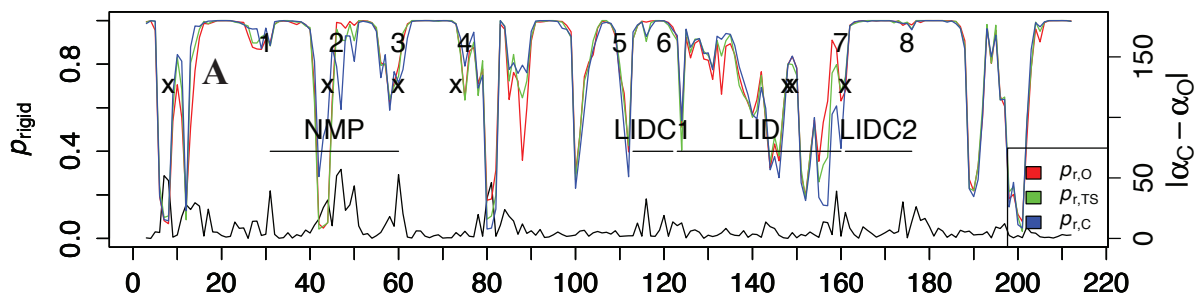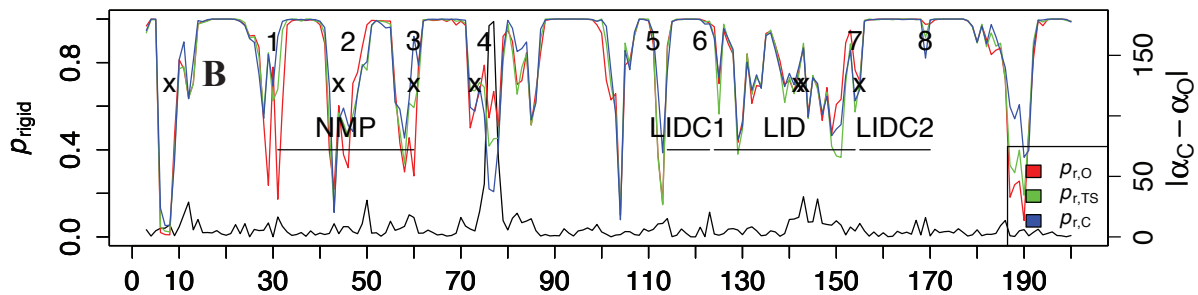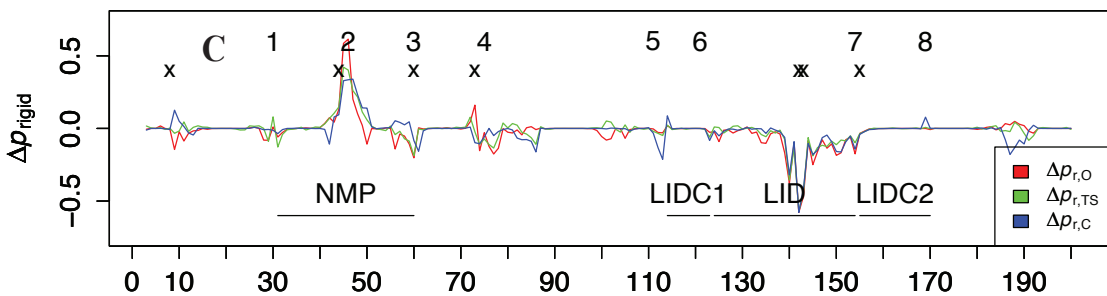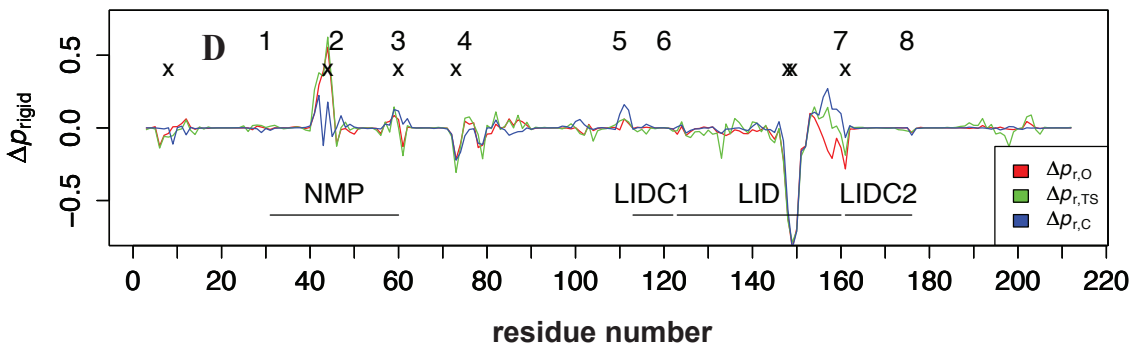

Supplement: Figure S5 — Small-scale backbone flexibility in key simulations. In each panel, p rigid,E for the pseudodihedral angle α i-1,i is defined as fraction of structures in ensemble E for which α is in the same rotamer (within 20°) as the “reference value,” which is the average of the O and C native state values if |Δα|<20°, where Δα = α C−α O. To simplify the calculation for residues with |Δα|≥20°, three reference values are used (O, C, and the midpoint), and the highest probability among those three is defined as p rigid. The black curve shows |Δα| for reference. Numbers 1–8 at the top mark the positions (respective central residues) of the eight hinges of Kern et al [14], and x's mark the 7 mutation sites. p rigid,TS is the average of the values for the closing and opening transitions. A: AKmeso; B: AKthermo. Panels C and D show the difference in p rigid (Δp rigid) from the appropriate wild-type simulation for AKthermo-7P and AKmeso+7P, respectively. (PDF) [file pcbi.1002103.s005.pdf]

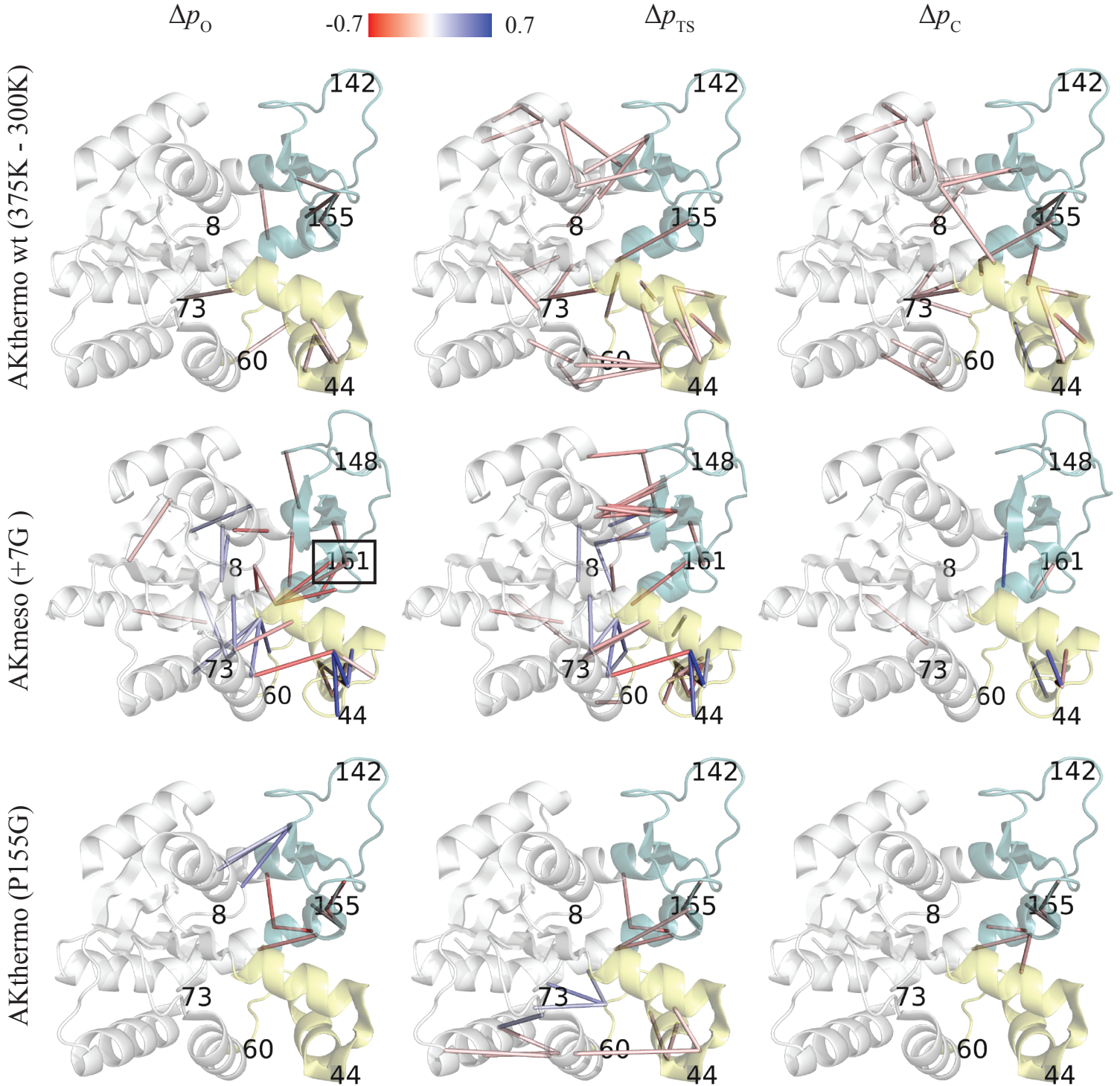

Supplement: Figure S6 — Changes in contact probabilities resulting from additional variants of AKthermo. Top row: AKthermo (375K-300K simulation); Second row: AKmeso (+7G-wt); Third row: AKthermo (P155G-wt). Otherwise, panels are labeled and colored as in Figure 5 of the main text. (PDF) [file pcbi.1002103.s006.pdf]

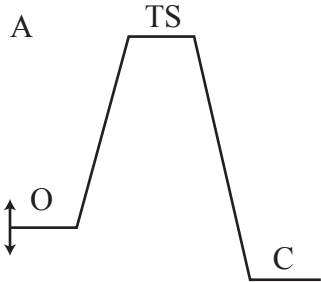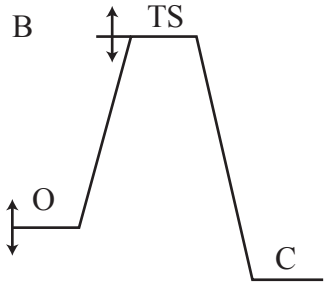

Supplement: Figure S7 — Thermodynamic and kinetic effects of perturbing different types of degrees of freedom. A: For a degree of freedom that is C-like in the TS, perturbations will primarily affect the free energy of O, and thus the TS-O free energy difference and the closing rate. B: For a degree of freedom that is O-like in the TS, perturbation will affect the free energies of O and TS similarly, modulating the TS-C free energy and by extension the opening rate. (PDF) [file pcbi.1002103.s007.pdf]

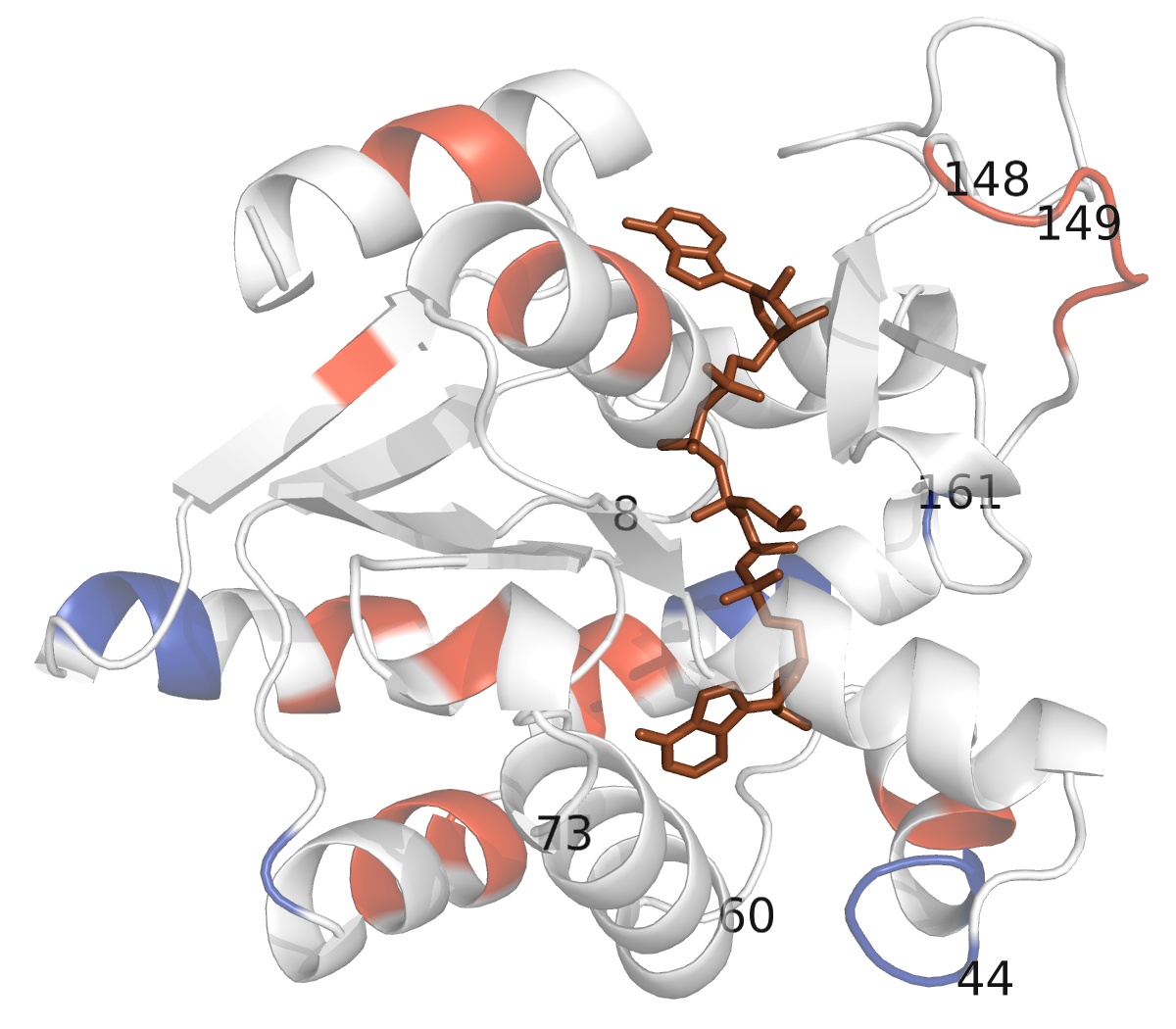

Supplement: Figure S8 — Differences in local structural entropy (LSE) between AKmeso and AKthermo. Red indicates residues for which LSE is lower by 0.15 or more in AKthermo; blue indicates that LSE is lower by 0.15 or more in AKmeso. Brown sticks indicate the substrate analog. (PNG) [file pcbi.1002103.s008.png]

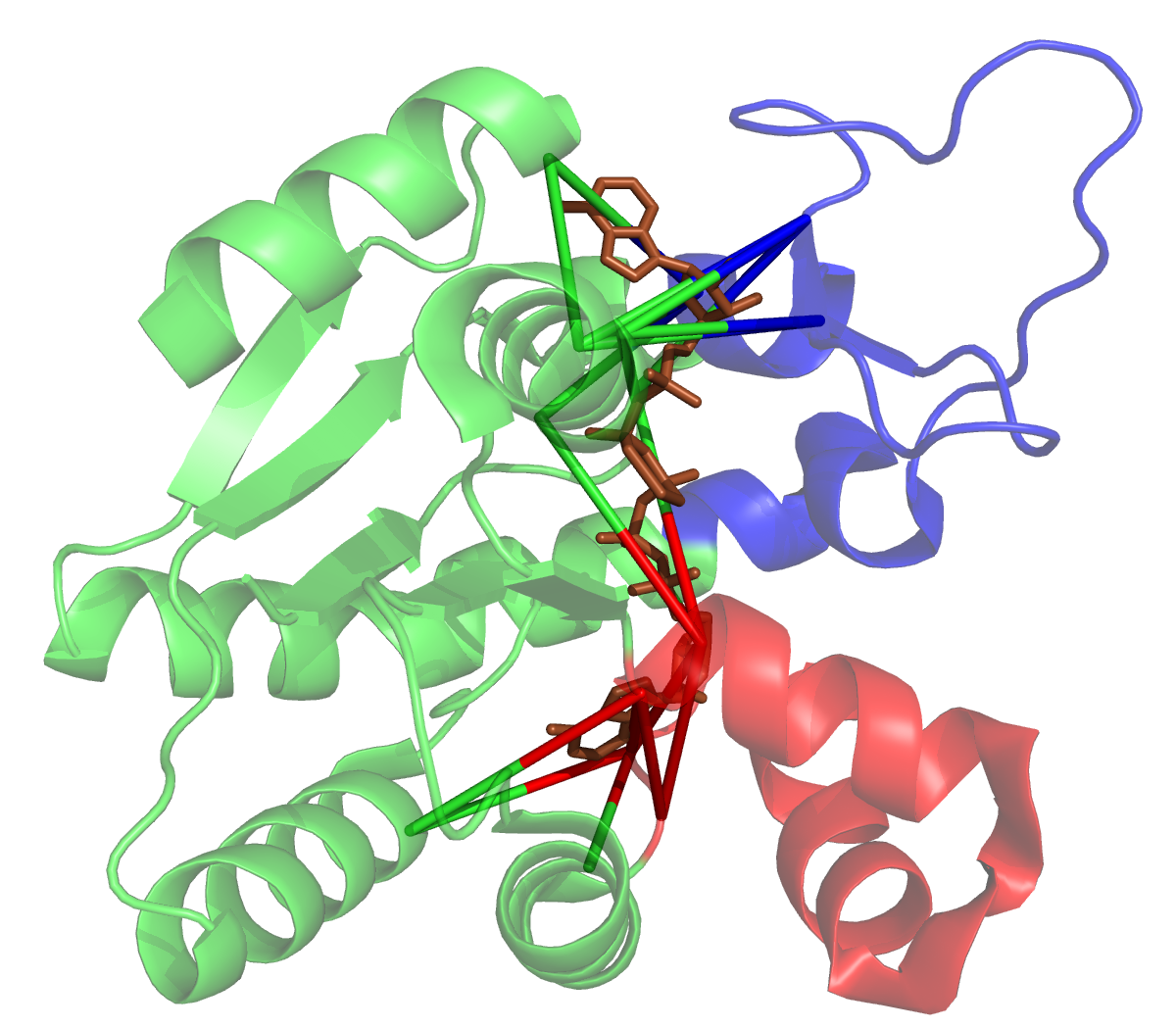

Supplement: Figure S9 — Ligand-mediated interactions mapped onto the closed Aquifex AK crystal structure (2RGX). CORE, LID, and NMP domains are shown in green, blue, and red, respectively. Ligand-mediated interactions are indicated by pseudo-bonds between the Cα atoms of involved residues. (PNG) [file pcbi.1002103.s009.png]

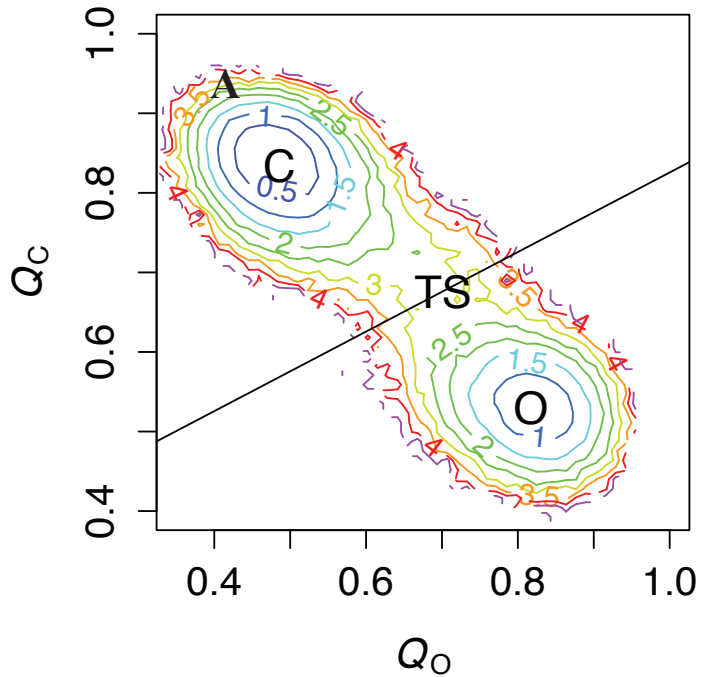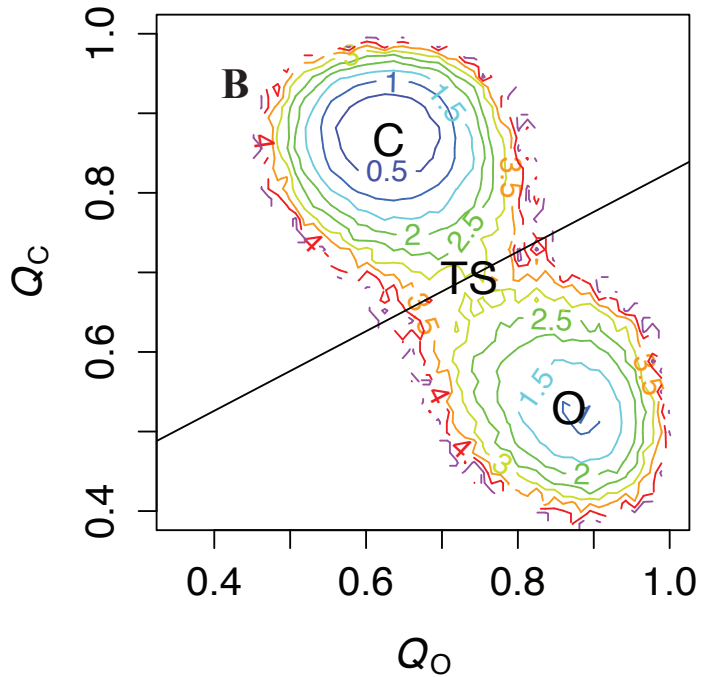

Supplement: Figure S10 — The bound AKmeso and AKthermo transitions in global contact reaction coordinates. Estimated potential of mean force (PMF) for AKmeso (A) and AKthermo (B) simulations based on projection of simulation data onto Q O and Q C reaction coordinates, where Q O(C) is the fraction of native contacts unique to the O(C) crystal structure. The diagonal lines show the transition separatrices calculated as in our previous work. Contours are spaced at 0.5 kcal/mol intervals in both PMFs. (PDF) [file pcbi.1002103.s010.pdf]

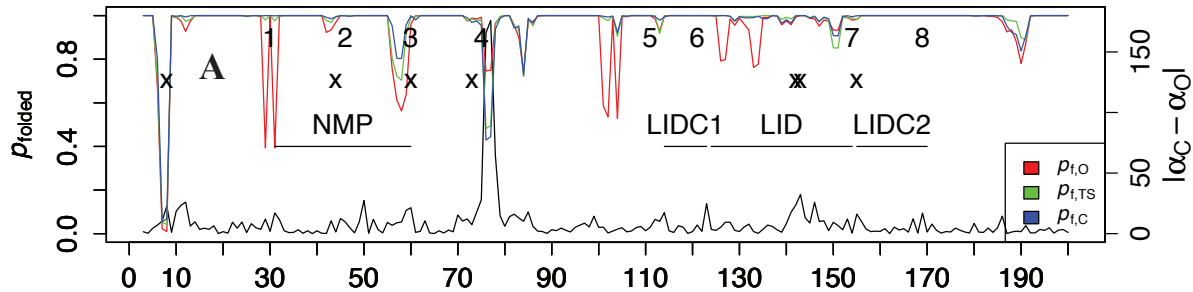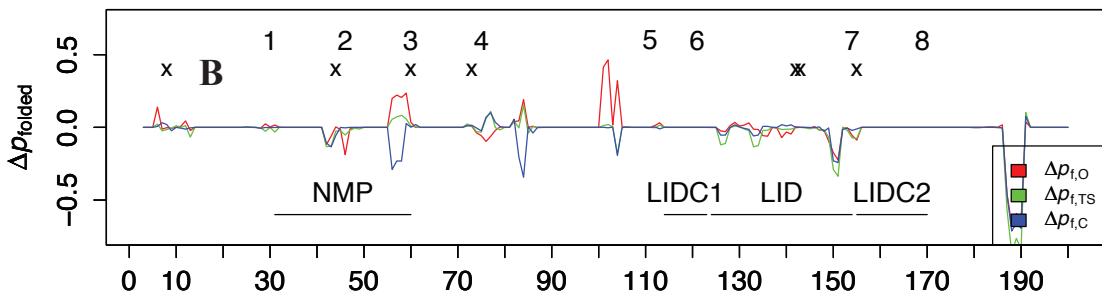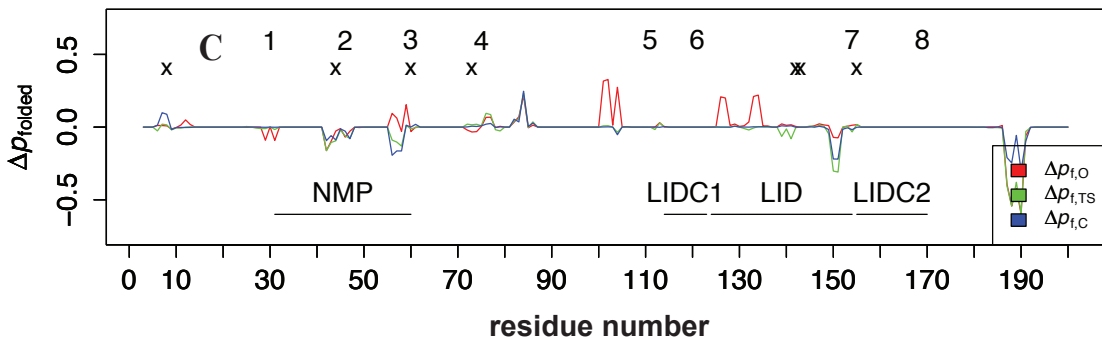

Supplement: Figure S11 — Local unfolding in different minimization variants of AKthermo. A: unminimized simulation; B and C: for unrestrained minimization and position-restrained minimization, respectively, Δp folded vs. the unminimized simulation. Panels are labeled as in Figure 3 of the main text. (PDF) [file pcbi.1002103.s011.pdf]

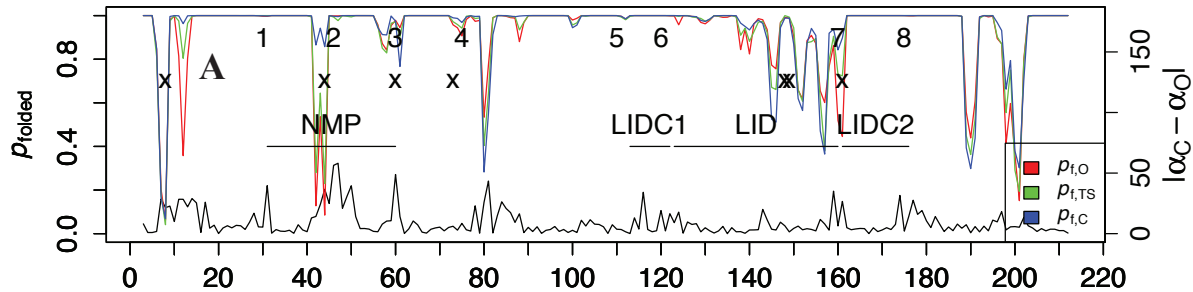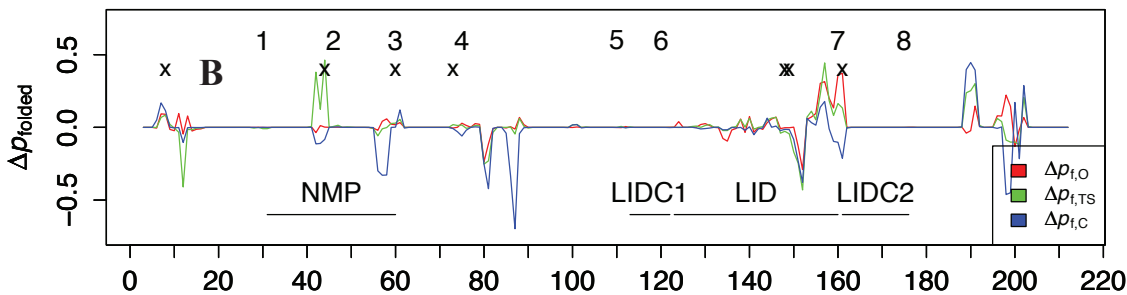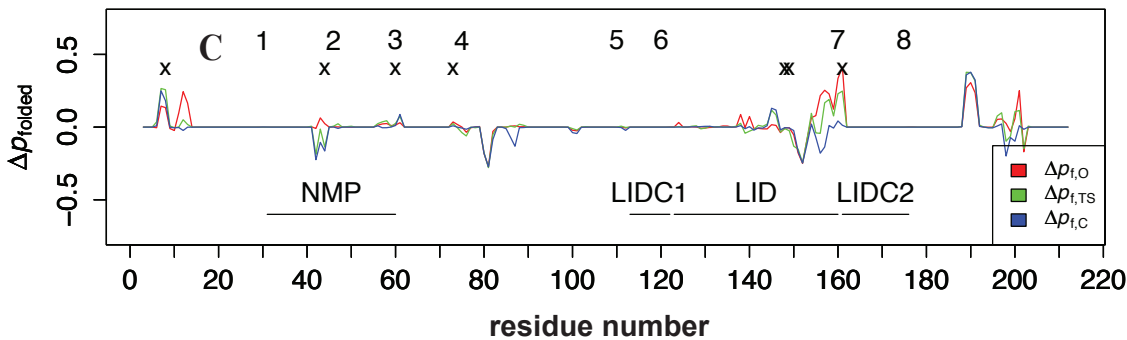

Supplement: Figure S12 — Local unfolding in different minimization variants of AKmeso. A: unminimized simulation; B and C: for unrestrained minimization and position-restrained minimization, respectively, Δp folded vs. the unminimized simulation. Panels are labeled as in Figure 3 of the main text. (PDF) [file pcbi.1002103.s012.pdf]

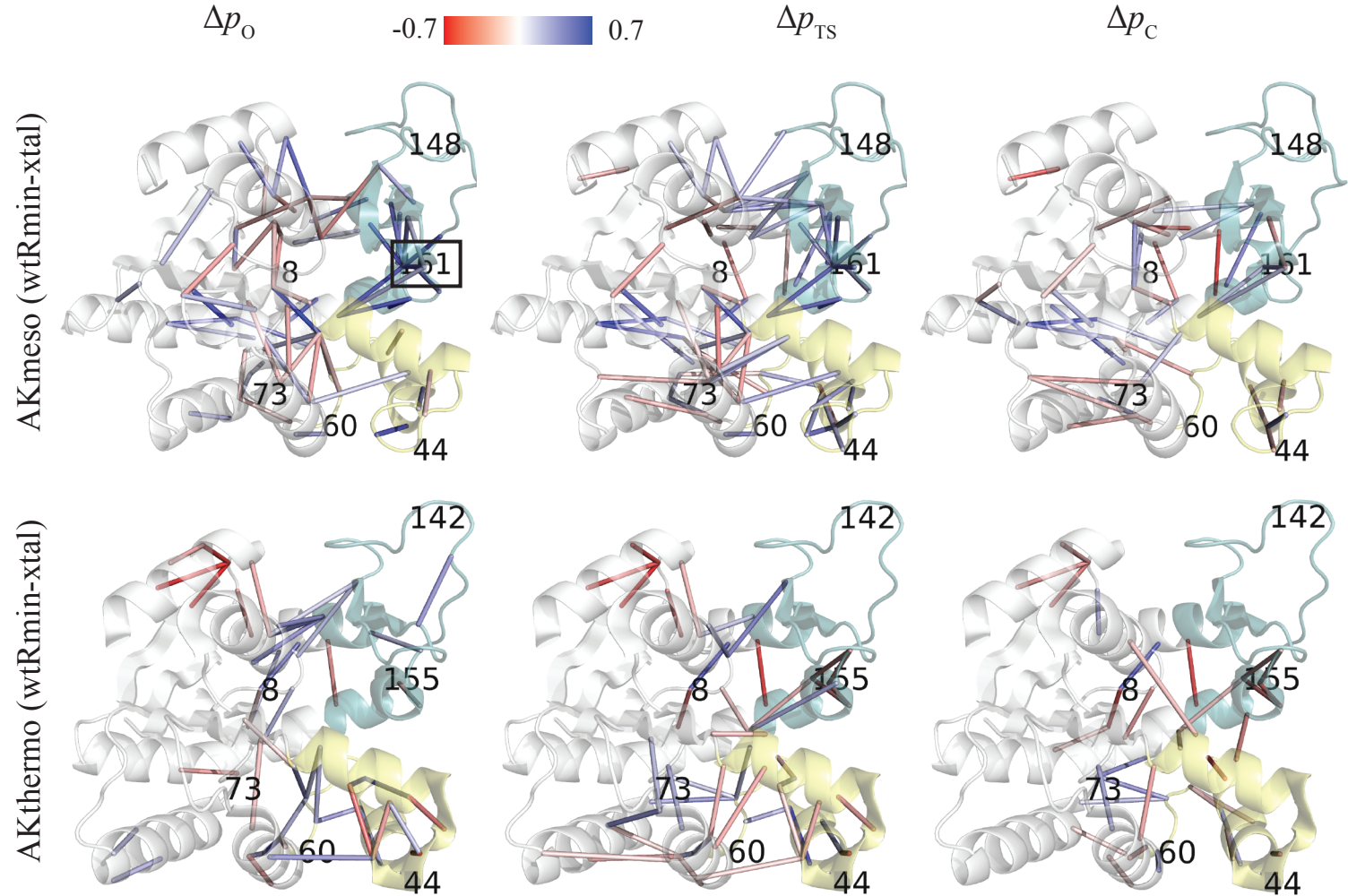

Supplement: Figure S13 — Changes in contact probabilities between crystal structure-based wild type simulations and wild-type simulations after position-restrained minimization. Panels are labeled and colored as in Figure 5 of the main text. (PDF) [file pcbi.1002103.s013.pdf]
