# Supplementary material for: Interconversion of Functional Motions between Mesophilic and Thermophilic Adenylate Kinases
Source: PLoS Comput Biol. 2011 Jul 14;7(7):e1002103. doi: 10.1371/journal.pcbi.1002103 (PMC3136430; doi:10.1371/journal.pcbi.1002103)
Supplement: Table S1 — Double-well Gō model parameters and statistics for simulations in this work. (DOC) [file pcbi.1002103.s014.doc]

Table S1: Double-well Gō model parameters and statistics for simulations in this work

| variant | −ε_O_ | *β*_mix_ | Δbar | *K*_eq_ | *p*_TP_ |
| --- | --- | --- | --- | --- | --- |
| M-wt | 66.0 | 0.0210 | 33.0 | 2.5 | 0.37 |
| T-wt | 68.0 | 0.0210 | 33.0 | 4.5 | 0.48 |
| T-wt-375K | 64.0 | 0.0210 | 33.0 | 2.3 | 0.48 |
| T-7P | 77.0 | 0.0210 | 33.0 | 2.9 | 0.42 |
| T+7G | 71.0 | 0.0210 | 33.0 | 2.6 | 0.38 |
| M+7P | 87.0 | 0.0224 | 31.0 | 4.4 | 0.34 |
| M-apo | 22.0 | 0.0301 | 23.0 | 3.2 | 0.32 |
| T-apo | 25.0 | 0.0301 | 23.0 | 3.0 | 0.50 |
| M+7G | 83.0 | 0.0210 | 33.0 | 2.3 | 0.32 |
| T-P155G | 70.0 | 0.0210 | 33.0 | 2.1 | 0.47 |
| T-P142G+P143G | 68.0 | 0.0210 | 33.0 | 3.4 | 0.54 |
| T-P8G | 72.0 | 0.0210 | 33.0 | 2.6 | 0.50 |
| T-P8G+P155G | 72.0 | 0.0210 | 33.0 | 4.6 | 0.43 |
| M-xtal | 97.0 | 0.0185 | 37.5 | 3.3 | 0.40 |
| T-xtal | 76.0 | 0.0231 | 30.0 | 4.3 | 0.45 |
| M-wt-min | 46.0 | 0.0257 | 27.0 | 4.4 | 0.49 |
| T-wt-min | 57.0 | 0.0204 | 34.0 | 3.2 | 0.44 |

T indicates AKthermo; M indicates AKmeso. ‘wt’ indicates wild type with position-restrained minimization (see methods), ‘xtal’ indicates simulation starting from the crystal structure, and ‘wt-min’ indicates unconstrained minimization. Δbar = ln(2)/*β*_mix_ is the approximate reduction of the barrier height in kcal/mol. Exponential averaging (potential mixing) parameters ε_O_ and *β*_mix_ are defined in our previous work [1] and Best et al. (2005) [2].

**References**

1. Daily MD, Phillips GN, Jr., Cui Q (2010) Many local motions cooperate to produce the adenylate kinase conformational transition. J Mol Biol 400: 618-631.

2. Best RB, Chen YG, Hummer G (2005) Slow protein conformational dynamics from multiple experimental structures: the helix/sheet transition of arc repressor. Structure 13: 1755-1763.
