# Supplementary material for: Interconversion of Functional Motions between Mesophilic and Thermophilic Adenylate Kinases
Source: PLoS Comput Biol. 2011 Jul 14;7(7):e1002103. doi: 10.1371/journal.pcbi.1002103 (PMC3136430; doi:10.1371/journal.pcbi.1002103)
Supplement: Table S2 — Positions of O and C wells for simulations in this work. (DOC) [file pcbi.1002103.s015.doc]

Table S2: Positions of O and C wells for simulations in this work

| variant | *rms*_O_ | *rms*_C_ | *Q*_O_ | *Q*_C_ |
| --- | --- | --- | --- | --- |
| M-wt | 2.79 | 2.15 | 0.81 | 0.83 |
| T-wt | 2.87 | 1.46 | 0.87 | 0.87 |
| T-wt-375K | 3.13 | 1.79 | 0.81 | 0.81 |
| T-7P | 3.18 | 1.6 | 0.84 | 0.85 |
| T+7G | 3.29 | 1.69 | 0.87 | 0.84 |
| M+7P | 3.08 | 2.3 | 0.78 | 0.83 |
| M-apo | 2.63 | 2.68 | 0.79 | 0.82 |
| T-apo | 2.6 | 2.6 | 0.88 | 0.87 |
| M+7G | 3.46 | 2.36 | 0.77 | 0.83 |
| T-P155G | 3.06 | 1.52 | 0.87 | 0.85 |
| T-P142G+P143G | 2.99 | 1.54 | 0.87 | 0.87 |
| T-P8G | 3.14 | 1.57 | 0.86 | 0.86 |
| T-P8G+P155G | 3.17 | 1.58 | 0.87 | 0.85 |
| T-wt-weak | 2.93 | 1.57 | 0.85 | 0.85 |
| M-xtal | 3.27 | 2.35 | 0.79 | 0.83 |
| T-xtal | 3.05 | 1.46 | 0.88 | 0.86 |
| M-wt-min | 3.25 | 2.11 | 0.81 | 0.82 |
| T-wt-min | 2.82 | 1.48 | 0.8 | 0.85 |

T indicates AKthermo; M indicates AKmeso. For consistency with the main text, *Q*_O_ and *Q*_C_ are here based on contacts unique to the crystal structures rather than the O-ensemble-characteristic and C-characteristic contact sets, which may differ between mutants.
