# Supplementary material for: Interconversion of Functional Motions between Mesophilic and Thermophilic Adenylate Kinases
Source: PLoS Comput Biol. 2011 Jul 14;7(7):e1002103. doi: 10.1371/journal.pcbi.1002103 (PMC3136430; doi:10.1371/journal.pcbi.1002103)
Supplement: Table S3 — Average ( r CM,core-nmp, r CM,core-lid) positions and standard deviations of the O ensemble for simulations in this work. (DOC) [file pcbi.1002103.s016.doc]

Table S3: Mean (*r*_CM,core-nmp_, *r*_CM,core-lid_) positions and standard deviations of the O ensemble for simulations in this work

|  | *r*_CM,core-lid_ (Å) | | | |  | *r*_CM,core-nmp_ (Å) | | | |
| --- | --- | --- | --- | --- | --- | --- | --- | --- | --- |
| simulation | mean | Δmean | sd | Δsd |  | mean | Δmean | sd | Δsd |
| M-wt | 30.9 |  | 2.2 |  |  | 22.6 |  | 0.3 |  |
| M+7P | 30.8 | -0.2 | 2.2 | 0.0 |  | 22.5 | -0.1 | 0.4 | 0.0 |
| M+7G | 33.2 | 2.3 | 3.9 | 1.7 |  | 22.5 | -0.1 | 0.4 | 0.0 |
| M-apo | 31.8 | 0.8 | 2.0 | -0.2 |  | 22.7 | 0.0 | 0.4 | 0.0 |
| M-xtal | 31.3 | 0.3 | 2.4 | 0.3 |  | 22.7 | 0.1 | 0.4 | 0.1 |
| M-wt-min | 29.0 | -1.9 | 2.2 | 0.0 |  | 22.4 | -0.2 | 0.3 | 0.0 |
| T-wt | 28.3 |  | 1.9 |  |  | 21.5 |  | 0.5 |  |
| T-wt-375K | 28.6 | 0.3 | 2.3 | 0.5 |  | 21.5 | 0.0 | 0.6 | 0.1 |
| T-7P | 28.0 | -0.3 | 2.4 | 0.5 |  | 21.6 | 0.2 | 0.5 | 0.1 |
| T+7G | 29.9 | 1.6 | 3.2 | 1.4 |  | 21.3 | -0.2 | 0.6 | 0.1 |
| T-apo | 29.7 | 1.4 | 1.7 | -0.2 |  | 21.3 | -0.2 | 0.6 | 0.1 |
| T-P155G | 27.9 | -0.4 | 2.1 | 0.2 |  | 21.5 | 0.0 | 0.5 | 0.0 |
| T-P142G+P143G | 28.0 | -0.3 | 2.0 | 0.2 |  | 21.5 | 0.0 | 0.5 | 0.0 |
| T-P8G | 28.8 | 0.5 | 2.7 | 0.8 |  | 21.5 | 0.0 | 0.5 | 0.0 |
| T-P8G+P155G | 29.3 | 1.0 | 3.0 | 1.1 |  | 21.4 | -0.1 | 0.5 | 0.1 |
| T-wt-weak | 28.4 | 0.1 | 2.1 | 0.2 |  | 21.5 | 0.0 | 0.5 | 0.0 |
| T-xtal | 28.8 | 0.4 | 2.3 | 0.4 |  | 21.7 | 0.2 | 0.6 | 0.1 |
| T-wt-min | 29.5 | 1.2 | 2.2 | 0.3 |  | 21.7 | 0.2 | 0.5 | 0.0 |

Variants are labeled as in Table S2. The standard deviations approximately measure the flexibility of NMP and LID rigid-body motions in the O ensemble. Δmean and Δsd are measured relative to the wild-type AKmeso or AKthermo simulation as appropriate. All simulations use position-restrained minimization (see methods), except M/T-xtal, which are based directly on the crystal structures, and M/T-wt-min, which are based on unrestrained minimization (see methods).
